# The combined influence of microbiome and soil environment contributes to the chemotype differentiation in *Atractylodes lancea*

**DOI:** 10.1002/imo2.70058

**Published:** 2025-10-09

**Authors:** Hongyang Wang, Zheng Peng, Chengcai Zhang, Chuanzhi Kang, Yan Zhang, Xiuzhi Guo, Yiheng Wang, Guang Yang, Zengxu Xiang, Li Zhou, Zhixian Jing, Dahui Liu, Sheng Wang, Luqi Huang, Lanping Guo

**Affiliations:** ^1^ State Key Laboratory for Quality Ensurance and Sustainable Use of Dao‐di Herbs, National Resource Center for Chinese Materia Medica China Academy of Chinese Medical Sciences Beijing China; ^2^ Key Laboratory of Biology and Cultivation of Herb Medicine Ministry of Agriculture and Rural Affairs Beijing China; ^3^ Dexing Research and Training Center of Chinese Medical Sciences Dexing China; ^4^ Pharmacy Faculty Hubei University of Chinese Medicine Wuhan China; ^5^ College of Horticulture Nanjing Agricultural University Nanjing China

**Keywords:** *Atractylodes lancea*, chemotype, climate, genotype, microbiome

## Abstract

*Atractylodes lancea*, a traditional Chinese medicinal herb, is divided into two chemotypes based on the production of volatile bioactive compounds: the geo‐authentic Maoshan chemotype (MSA, rich in atractylon and atractylodin) and the non‐authentic Hubei chemotype (HBA, dominated by hinesol and β‐eudesmol). However, the mechanisms underlying the differentiation of these chemotypes remain poorly understood. By sequencing analysis of wild and cultivated *A. lancea* samples from multiple provinces in China, we revealed that the genetic divergence into Maoshan‐Dabie Mountains group (MA) and Qinling‐Taihang Mountains group (SA) was occurred along altitudinal and climatic gradients, but the chemotype was not solely genetically determined. Interestingly, both MA and SA genotypes in the MSA‐favorable or HBA‐conducive soil could develop into the MSA or the HBA chemotype, respectively, indicating a role of the soil microbiome. Critically, specific rhizosphere microbiomes were indicated as key mediators‐core in this process, including *Streptomyces* in MSA formation and *Paenibacillus* in HBA formation. Shared endophytic core genera, such as *Rhodococcus*, *Ralstonia*, *Sphingomonas*, and *Bradyrhizobium*, further contributed to this divergence through species‐level functional variation. Using piecewise structural equation modeling, we further confirmed that altitude, climate, and soil properties directly or indirectly influenced the chemotype formation via genotype–microbiome interactions. Taken together, this study highlights the central role of soil and microbes in the chemotype differentiation of *A. lancea* and provides new insights into its underlying mechanisms. The regulatory role of microbes in the production of volatile bioactive compounds offers a theoretical foundation for the microbial breeding strategies to improve medicinal quality and clinical efficacy.

## Hightlights

- The genetic differentiation of the MA and SA genotypes of *Atractylodes lancea* takes place along gradients of altitude and climate.
- *Atractylodes lancea* undergoes chemotype differentiation influenced by soil chemistry and microbiota, rather than being determined by its genotype.
- Rhizosphere and endophytic core microbes drive the chemotype differentiation of *Atractylodes lancea*.

## Graphical abstract

Genetic differentiation between the Maoshan‐Dabie Mountains group (MA genotype) and Qinling‐Taihang Mountains group (SA genotype) of *Atractylodes lancea* occurs along altitude and climatic gradients. High altitudes and their corresponding climatic conditions favor the formation of SA, while low altitudes and their corresponding climatic conditions favor the formation of MA. The chemotype differentiation of the geo‐authentic Maoshan chemotype (MSA, rich in atractylon and atractylodin) and the non‐authentic Hubei chemotype (HBA, dominated by hinesol and β‐eudesmol) in *A. lancea* is influenced by soil chemistry and microbiota, rather than being determined by its genotype. Rhizosphere and endophytic core microbes are the driving forces behind the chemotype differentiation of *A. lancea*.

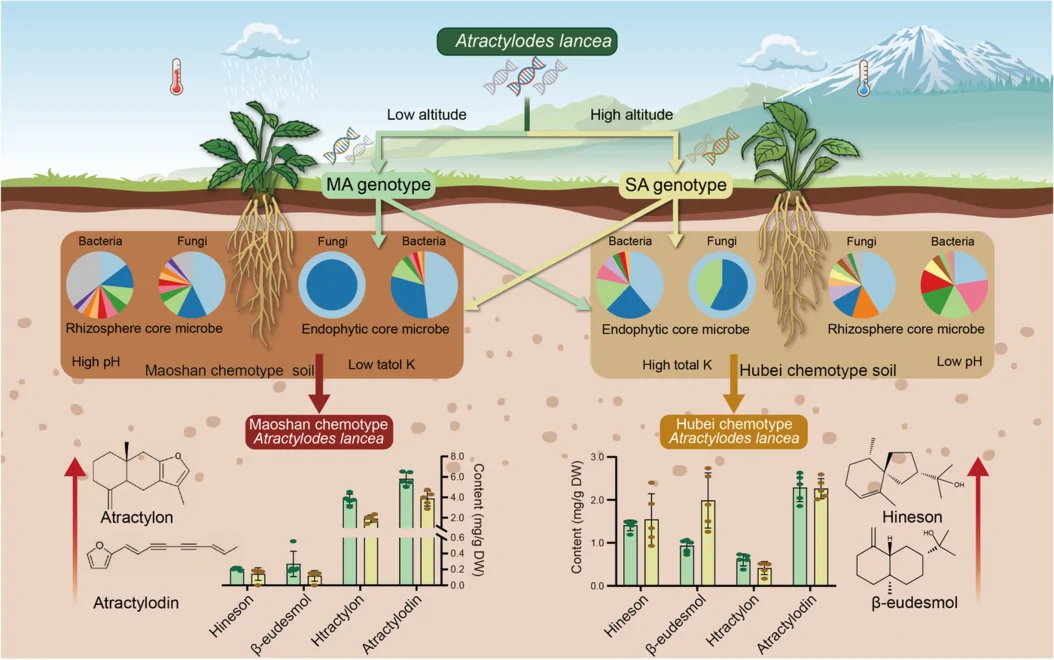

## 1 INTRODUCTION

*Atractylodes lancea*, a perennial herb of the *Atractylodes* in the Compositae, is widely distributed in China, primarily in Jiangsu, Anhui, Hubei, Henan, Shaanxi, Hebei, and Inner Mongolia. The *A. lancea* rhizome (ALR), known as “maocangzhu” or “beicangzhu” has been used in traditional Chinese medicine for thousands of years [1]. *A. lancea* exhibits notable diversity, particularly in the volatile oil composition, which varies by region and even within the same area [2]. Guo et al. [3, 4] identified two main chemotypes: (1) the Maoshan Cangzhu chemotype (MSA), found in Jiangsu, Shandong, Hebei, and northern Henan, contains lower volatile oil content, with a predominance of atractylon and atractylodin; and (2) the Hubei Cangzhu chemotype (HBA), found in regions like Hubei, Anhui, Shaanxi, and southern Henan, characterized by high volatile oil content of hinesol and β‐eudesmol, but low level of or no atractylon or atractylodin. Thus, the ratio of hinesol, β‐eudesmol, atractylon, and atractylodin in volatile oil plays a key role in such classification. Secondary metabolites, including volatile oils, are critical to the medicinal efficacy of Dao‐di herbs, contributing to their distinct chemotypes and superior therapeutic effects [5]. A chemotype is an ecotype variant driven by phenotypic plasticity, reflecting environmentally influenced genetic expression [6]. Zhang et al. [7] used genome resequencing to analyze *A. lancea* populations, and identified key genes involved in medicinal quality and genotype classification based on the features of gene structures: the Maoshan‐Dabie Mountains Group (MA), the North Yanshan Mountains Group (NA), and the Qinling‐Taihang Mountains Group (SA). Their study highlighted that “high‐quality” *A. lancea* represents a chemotype shaped by ecological adaptation to environmental stresses. Volatile oils, especially hinesol, β‐eudesmol, atractylon, and atractylodin, are key active ingredients of *A. lancea*, responsible for its medicinal effects including drying dampness, invigorating the spleen, and providing antibacterial and anti‐inflammatory benefits [8, 9, 10]. Guo et al. [11] conducted regression analyses to identify climate factors that affect volatile oil content, and revealed that temperature, in interaction with precipitation, is the primary determinant. The Maoshan habitat, characterized by high temperatures and precipitation, is associated with high‐temperature stress, influencing the formation of its volatile oil composition. In addition, Wang et al. [12, 13] showed that heat and drought stress inhibited the growth of *A. lancea* and altered root‐associated microbial communities. To withstand such environmental stress, *A. lancea* could recruit specific microbiota within its rhizosphere and rhizomes, thereby enhancing the content of its four primary volatile oils.

The finding of endophytic fungus *Taxomyces andreanae* that produces paclitaxel and other taxane compounds with antitumor activities in the phloem of *Taxus brevifolia* sparked a trend of isolating endophytes from medicinal plants [14]. Subsequently, endophytic bacteria in medicinal plants can promote the production and accumulation of active substances in host plants, which is correlated with the quality of host plants [15]. Many studies have confirmed that endophytes drive the biosynthesis and accumulation of active compounds in both *A. lancea*. The endophytes *Gilmaniella* sp. and *Pseudomonas fluorescens* promote the production of sesquiterpene active ingredients such as hinesol, β‐eudesmol, attractylone, β‐sesquiphellandrene, and Zingiberene [16, 17, 18]. Wang Hongyang discovered that fungi primarily enhance the levels of atractylone and atractylodin in the rhizomes of *A. lancea*, whereas bacteria mainly facilitate the accumulation of hinesol and β‐eudesmol [19]. On the other hand, plant metabolites can also affect microbial community composition [20, 21]. Brachi B et al. [22] used genome‐wide association studies to explore how *Arabidopsis thaliana* genotypes regulate core microbiota to affect plant adaptability. Their findings emphasized the role of genotypes in modulating microbiota for improved plant performance. This interdependence highlights the critical role of microbial communities in determining the quality of traditional Chinese medicine. Additionally, Yang et al. [23] found different growth patterns of rhizosphere microorganisms and flavonoid composition between wild and cultivated *Scutellaria baicalensis*. Similarly, healthy plant roots of *Salvia miltiorrhiza* recruited some potentially beneficial bacteria, such as *Pseudomonas* to effectively increase seedling growth, crop yield, and the content of effective medicinal components [24]. Su et al. [25] examined the role of the microbiome in the authenticity of *Citrus reticulata* “Chachi” demonstrating that soil properties and microbiota influenced monoterpene synthesis in citrus peel, paving the way for improving fruit quality through fertilization and microbiota management.

In conclusion, it is evident that the accumulation of volatile oils in ALR is influenced by genetic, abiotic, and especially microbial factors. However, the underlying mechanisms and relationships between environmental factors (climate, soil, and microorganisms) and genetic factors, in shaping the chemotypes of *A. lancea* remain elusive. In this study, controlled experiments and multivariate statistical analyses were used to investigate how environmental factors and microorganisms shape genotype and chemotype differentiation. We found that the genetic differentiation of MA and SA genotypes of *A. lancea* occurs along gradients of altitude and climate. *A. lancea* undergoes chemotype differentiation influenced by soil chemistry and microbiota, rather than being determined by its genotype. The rhizosphere and endophytic core microbes drive the differentiation of *A. lancea* chemotypes. These findings will offer a theoretical foundation for developing microbial fertilizers to enhance active ingredients production in medicinal plants, with significant implications for Traditional Chinese Medicine Ecological Agriculture.

## 2 RESULTS

### Genetic background and chemotype analysis of *A. lancea*

**Figure 1:**
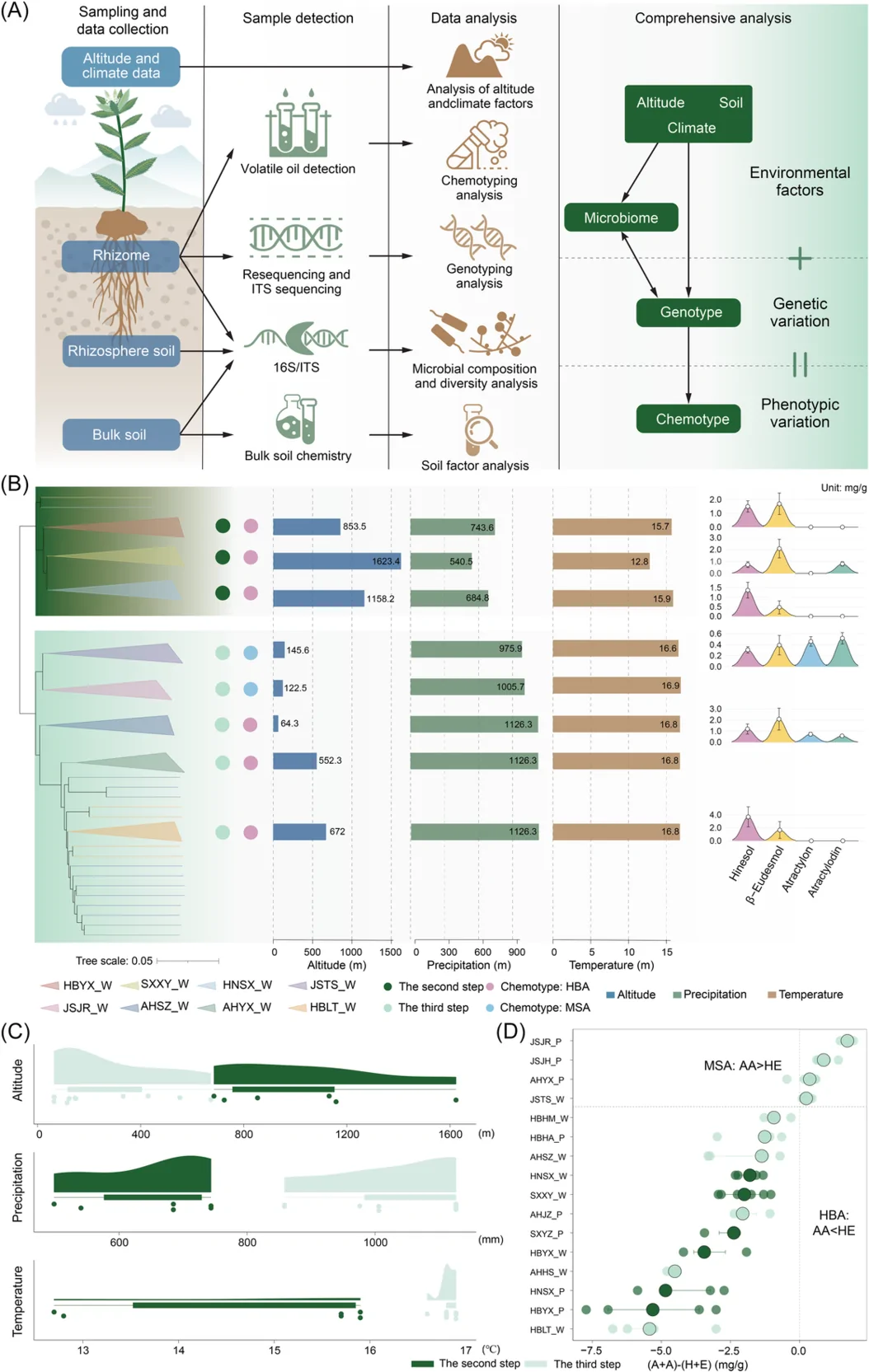
Chemotype distribution of geographic and genetic groups. (A) Flow diagram of sample collection and data analysis. (B) Phylogenetic clustering of genotypes mirrors geographic origin. Environmental parameters (altitude, precipitation, and temperature) are concordant with genetic structure. In contrast, the distribution of four major chemical components—hinesol, β‐eudesmol, atractylon, and atractylodin—shows no significant alignment with geography or genotype (*n* = 5). Error bars indicate mean ± standard deviation (SD). HBYX, Yunxian, Hubei Province; SXXY, Xunyi, Shaanxi Province; HNSX, Songxian, Henan Province; JSTS, Tangshan, Jiangsu Province; JSJR, Jurong, Jiangsu Province; AHSZ: Shizigang, Anhui Province; AHYX, Yuexi, Anhui Province; HBLT, Luotian, Hubei Province. W, wild *Atractylodes lance*. (C) Environmental variables differ significantly between geographic steps: the second step exhibits higher altitude but lower temperature and precipitation. Shaded areas represent mean ± SD. (D) The difference between the combined contents of atractylon and atractylodin (AA) versus that of hinesol and β‐eudesmol (HE) defines two chemotypes: Hubei chemotype (HBA) and Maoshan chemotype (MSA). Large black circles indicate group medians, with whiskers representing interquartile ranges (*n* = 5).

A total of 187 wild and cultivated ALRs were collected from 16 populations across five provinces in China, namely Jiangsu, Anhui, Hubei, Henan, and Shaanxi (Figure 1A and Table S1). Among these, 109 wild samples were utilized for resequencing, resulting in the identification of 11,522,685 high‐quality single‐nucleotide polymorphisms. Phylogenetic analysis using the neighbor‐joining method revealed that these wild samples could be divided into two distinct genotypes: MA, mainly from Jiangsu, Anhui, the eastern part of Hubei; and SA, from Henan, and Shaanxi, and the western part of Hubei (Figure 1A,B and Table S1). Internal transcribed spacer (ITS)‐based phylogenetic analysis of the remaining 78 wild and cultivated samples produced consistent genotype assignments (Figure S1). Further analysis showed that genotype distribution is closely associated with topography: MA is predominantly distributed in low‐altitude regions of the third geographic step (64.30–671.98 m), whereas SA occurs in high‐altitude regions of the second geographic step (684.43–1623.45 m). These two altitudinal zones have markedly different climatic conditions, with less rainfall and lower temperatures in the high‐altitude areas, while more rainfall and higher temperatures in the low‐altitude areas (Figure 1B,C). Therefore, the distribution of these two genotypes (MA and SA) shows a clear separation (PC1 = 90.6%), which corresponds to the environmental gradient between China's Second and Third geographic step (Figure S2A,B).

Based on the composition and relative proportions of volatile oil components, the samples were classified into two chemotypes: MSA and HBA (Figure 1D), consistent with previous study [2]. MSA chemotype is characterized by a dominance of atractylon and atractylodin, which includes the wild and cultivated samples from Jiangsu as well as the cultivated samples from Anhui. While HBA chemotype is dominated by hinesol and β‐eudesmol, which includes the wild and cultivated samples from Anhui, Hubei, Henan, and Shaanxi. These results indicate that geographic distribution determines the SA genotype in high‐altitude second‐step areas and the MA genotype in low‐altitude third‐step areas. However, the MA genotype contains both MSA and HBA chemotypes (Figure 1B and Figure S2B).

### Analysis of microbial diversity in bulk soil, rhizosphere, and rhizome of *A. lancea*

**Figure 2:**
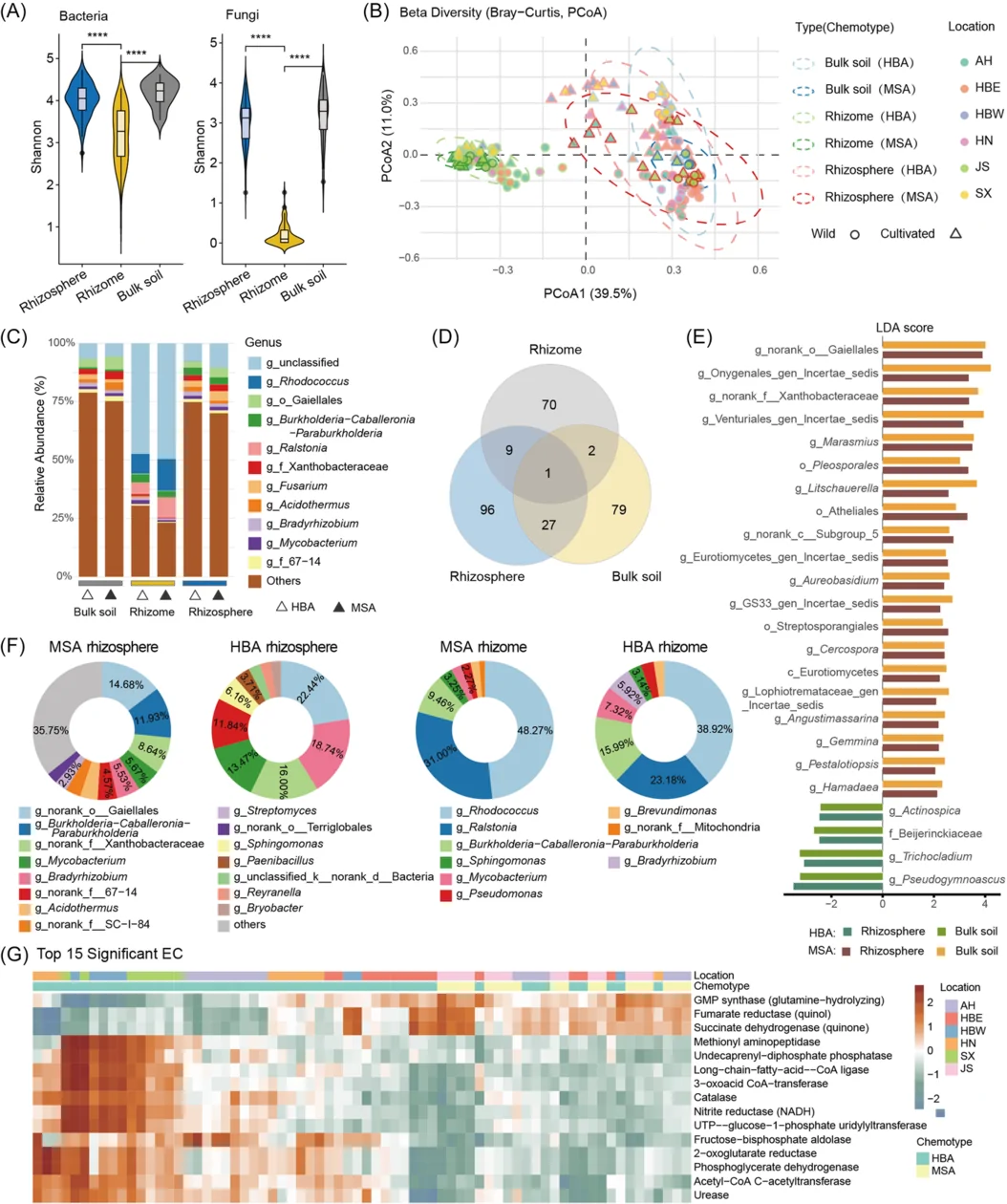
Analysis of microbial community diversity, composition, and functional potential in bulk soil, chemotype rhizome, and rhizosphere. (A) Boxplots showing alpha diversity (Shannon index) of bacterial (left) and fungal (right) communities in rhizosphere and bulk soil. ****p < 0.0001 (Wilcoxon test). (B) Principal Coordinates Analysis (PCoA) based on microbial Bray–Curtis dissimilarity. Symbols are shaped by cultivation status (wild: circles; cultivated: triangles) and colored by location, with 95% confidence ellipses matching the color of scatter points (blue: bulk soil; green: rhizome; red: rhizosphere; light/dark shades represent HBA and MSA chemotypes, respectively). (C) Stacked bar plots showing the percentage composition of the top 12 microbial genera in bulk soil, rhizome, and rhizosphere across different chemotypes. (D) Venn diagram depicting the shared differentially abundant microbial genera among rhizome, rhizosphere, and bulk soil compartments, with numbers indicating genus counts at the genus level. (E) Linear discriminant analysis effect size (LEfSe) showing genera commonly differentially abundant between HBA (rhizosphere: dark green; bulk soil: light green) and MSA (rhizosphere: dark orange; bulk soil: light orange) chemotypes (LDA score > 2, *p*adj < 0.05). (F) Pie charts of core bacterial genera in rhizosphere (left) and rhizome (right) for MSA and HBA chemotypes. (G) Heatmap of the top 15 significantly enriched Enzyme Commission (EC) functional categories, where color intensity represents row‐normalized values across samples. AH, Anhui Province; HBE, the eastern part of Hubei Province; HBW, the western part of Hubei Province; HN, Henan Province; JS, Jiangsu Province; SX, Shaanxi Province.

To minimize the effects of sequencing depth on alpha and beta diversity measure, the numbers of sequences for bacteria and fungi were rarefied to 13,262 and 13,919, which still yielded average Good's coverage of 99.85% and 99.86%, respectively. α‐diversity analysis revealed extremely significant differences in Shannon and Chao1 indices between habitats for both bacteria and fungi (*p* < 0.0001): bacterial diversity was highest in rhizosphere soil and bulk soil, followed by rhizome; while fungal diversity was significantly higher in rhizosphere soil and bulk soil than that in rhizome (Figure 2A and Figure S3). We found that the microbial community structure of rhizosphere soil was similar to that of bulk soil but significantly different from that of rhizome (PCoA1 = 39.5%, Figure 2B). Notably, genotype had a stronger effect on microbial community differentiation than chemotype, particularly in the divergence of endophytic fungal communities: endophytic fungi (rhizome samples) showed a complete separation under genotypic grouping (MA vs. SA) (Figure S4A). Additionally, geographic location (e.g., Jiangsu, Shaanxi) showed partially differentiated community structure, and wild (circles) versus cultivated (triangles) samples exhibited a trend of partial separation (Figure S4B).

### Microbial composition and differences between two *A. lancea* chemotypes

At the genus level, microbial communities varied by sample type (bulk soil, rhizome, rhizosphere) and chemotype (HBA/MSA). Unclassified genera (g_unclassified) exceeded 50% in rhizomes, while bulk soil and rhizosphere were dominated by “Others” (~75%) with low known genus abundance. Among classified taxa, *Rhodococcus* and *Ralstonia* were prominent in rhizomes, with potential treatment differences (Figure 2C). Results indicate sample type‐specific genus composition, with rhizome communities distinct from bulk soil/rhizosphere. Linear discriminant analysis effect size (LefSe) analysis (LDA > 2, *p* < 0.05) identified microbial genera with significant differences between the two chemotypes across bulk soil, rhizome, and rhizosphere samples (Table S2). To screen for mechanistic microbes driving chemotype differences under natural conditions and validate their synchronous variation in the rhizosphere, we focused on shared differential genera between bulk soil and rhizosphere. These shared 27 genera may represent key microbial markers that regulate host chemotype differentiation (Figure 2D). The LefSe LDA score plot showed microbial genera with consistent differential trends between HBA and MSA in both rhizosphere and bulk soil. Specifically, the bacterium O*_*Gaiellales and the fungus *Onygenales*_*gen*_*Incertae*_*sedis* were co‐enriched in the MSA rhizosphere and bulk soil, while the fungus *Pseudgymnoascus* and *Trichocladium*, and the bacterium f_Beijerinckiaceae and *Actinospica* were co‐enriched in HBA. These results indicate that the MSA chemotype exhibits a greater number of consistently differential genera with higher LDA scores (Figure 2E). Based on the stability of chemotype phenotypes, we focused on analyzing chemotype‐specific core microbial communities: O_Gaiellales (MSA 14.68%, HBA 22.44%) was dominant in both MSA and HBA rhizosphere, followed by *Burkholderia‐Caballeronia‐Paraburkholderia* (*B‐C‐P*) in MSA, but not in HBA. *Streptomyces* was exclusively found in MSA, whereas *Paenibacillus* and *Sphingomonas* were unique to HBA (Figure 2F). Unclassified fungi were dominant in the rhizosphere of MSA and HBA (Figure S5). In the rhizome, the composition of endophytic core bacteria of the two chemotypes was relatively similar, mainly including *Rhodococcus*, *Ralstonia*, *B‐C‐P*, *Sphingomonas*, and so on (Figure 2F). However, significant differences were observed in the endophytic core fungi at the amplicon sequence variant (ASV) level. Specifically, MSA was exclusively comprised of ASV6 (100%), whereas HBA was composed of both ASV6 (57.81%) and ASV1 (42.19%) (Figure S5). Although differential rhizosphere microbial genera existed between MSA and HBA, no core microbial genus in MSA showed consistently higher abundance than that of HBA among all samples (Figure S6), with only microorganisms involved in metabolic pathways such as GMP synthase (glutamine‐hydrolyzing), fumarate reductase (quinol), and succinate dehydrogenase (quinone) in MSA (Figure 2G).

### Effects of indigenous microbial communities and soil chemical properties on the chemotype formation

**Figure 3:**
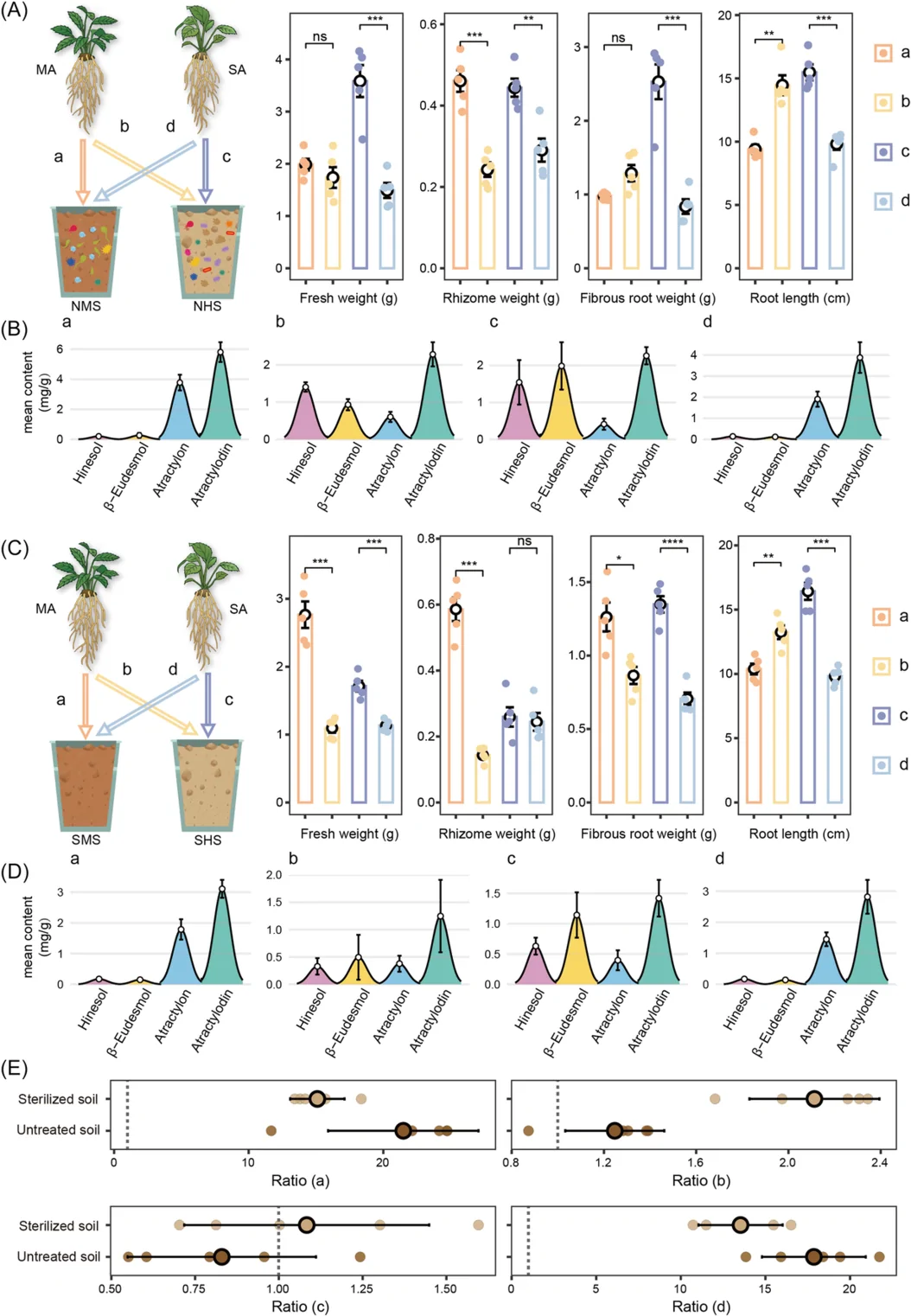
Chemotype performance and biomass traits of different chemotype plants grown in different soils. (A) Chemotype and biomass performance of different chemotype plants under untreated soil conditions. Schematic: a, MA in untreated soil from MSA region (NMS); b, MA in untreated soil from HBA region (NHS); c, SA in untreated soil from HBA region (NHS); d, SA in untreated soil from MSA region (NMS). Bar plots show fresh weight, rhizome weight, fibrous root weight, and root length (*n* = 10). Data are presented as mean ± SD, with significance determined by Student's *t*‐test (two‐sided). (B) Content of four characteristic volatile oil components (hinesol, β‐eudesmol, atractylon, and atractylodin) in plants grown under untreated soil conditions (*n* = 5). Panels a–d correspond to the soil–chemotype combinations in (A). Peak height indicates content, with error bars showing mean ± SD. (C) Chemotype and biomass performance of different chemotype plants under sterilized soil treatments. Schematic: groupings a–d follow the same designation as in (A). Bar plots show fresh weight, rhizome weight, fibrous root weight, and root length (*n* = 10). Data are presented as mean ± SD, with significance determined by Student's *t*‐test (two‐sided). ns, not significant; *p < 0.05; **p < 0.01; ***p < 0.001. (D) Content of characteristic volatile oil components under sterilized soil conditions (*n* = 5). Panels a–d correspond to the soil‐chemotype combinations in (C). Peak height indicates content, with error bars representing mean ± SD. (E) Ratios of (atractylon + atractylodin)/(hinesol + β‐eudesmol) across sterilized (top) and untreated (bottom) soils. Each point represents one biological replicate (*n* = 5); horizontal lines indicate means and error bars represent ± SD.

To investigate the role of soil properties and microorganisms in chemotype differentiation, we performed the greenhouse experiment of indigenous microorganisms to inoculate MA and SA, and explored the role of genotype, indigenous microorganisms, and soil property interaction in the chemotype formation of *A. lancea*. The experimental results of planting MA and SA in untreated soil (Figure 3A) showed that MA inoculated with untreated soil from MSA region (NMS) had significantly higher rhizome weight than untreated soil from HBA region (NHS) (*p* < 0.001) and slightly increased seedling fresh weight. In contrast, SA inoculated with NHS exhibited higher seedling fresh weight, rhizome weight, fibrous root weight, and longer root length than those with NMS (*p* < 0.01 or 0.001), demonstrating the adaptability to soil microenvironment. We also observed low hinesol and β‐eudesmol, but high atractylon and atractylodin in both MA and SA inoculated with NMS, promoting MSA formation (Figure 3B). Conversely, MA and SA inoculated with NHS yielded high hinesol and β‐eudesmol but low atractylon and atractylodin, favoring HBA formation (Figure 3B). These results indicate that soil microenvironment can strongly influence the chemical profiles of *A. lancea*. When growing in the sterilized soil from MSA region (SMS) or HBA region (SHS), MA showed higher seedling fresh weight (*p* < 0.001), rhizome weight (*p* < 0.001), and fibrous root weight (*p* < 0.05) in SMS than that in SHS; while SA exhibited higher fresh weight (*p* < 0.001), fibrous root weight (*p* < 0.001), and longer root length (*p* < 0.001) in SHS than that in SMS (Figure 3C). Although all four volatile oils were still produced by *A. lancea* growing in sterilized soil, the volatile oil production was largely restricted comparing with that growing in untreated soil (Figure 3D,E). This suggests that indigenous microbial influence, rather than genotype, plays a role in chemotype differentiation. Taken together, these results emphasize the pivotal role of indigenous microorganisms and soil chemical properties in shaping *A. lancea* chemotypes, supporting the microbial‐assisted breeding strategies and optimized cultivation.

### The functional verification of rhizosphere and endophytic core microorganisms in the differentiation of MSA and HBA

**Figure 4:**
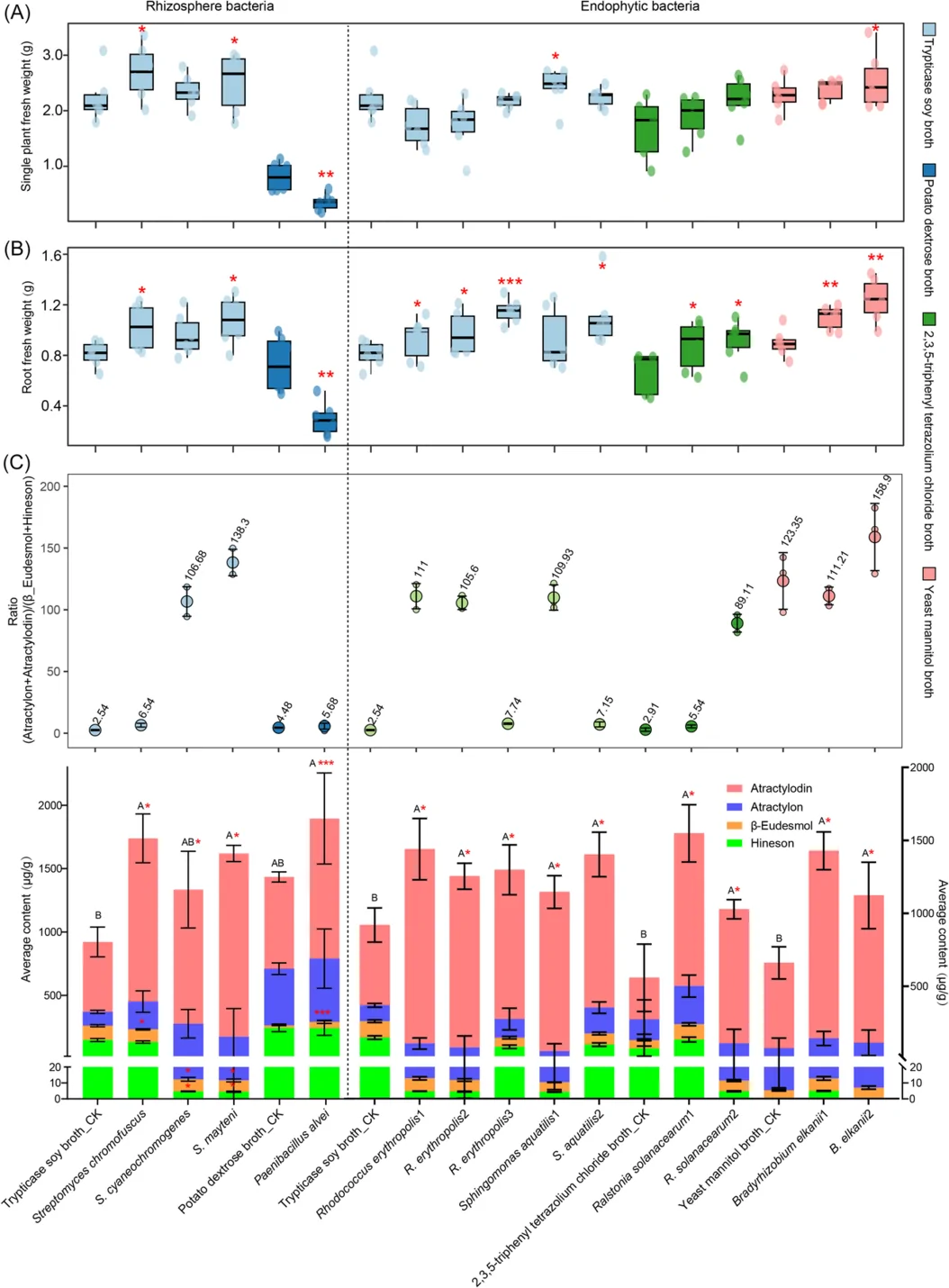
Effects of rhizosphere and endophytic core bacterial treatments on plant biomass and metabolite ratio. (A) The single plant fresh weight (*n* = 6). Data are presented as mean ± SD, with significance determined by Student's *t*‐test (two sided). (B) The root fresh weight (*n* = 6). Data are presented as mean ± SD, with significance determined by Student's *t*‐test (two sided). (C) Forest plot (top) showing the ratio of (atractylon + atractylodin)/(hinesol + β‐eudesmol) in rhizosphere bacteria (left) and endophytic bacteria (right) treatments, with error bars representing mean ± SD; bar plots showing the sum of four main volatile oils across treatments (*n* = 3). Data are presented as mean ± SD, with significance determined by Student's *t*‐test (two sided). Statistical significance was determined by unpaired *t*‐test (*p < 0.05, **p < 0.01, ***p < 0.001). “A”, “B,” and “C” respectively represent the significance of the sum of four volatile oils at the *p* < 0.05.

To further identify the rhizosphere and endophytic core microorganisms that regulate the chemotype differentiation of *A. lancea*, we performed a Spearman correlation analysis and conducted another greenhouse experiment utilizing core microorganisms that significantly correlated with volatile oil components. The Spearman correlation heatmap analysis unveiled that the MSA‐exclusive rhizosphere core bacterial genera *Streptomyces* was significantly negative correlated with both hinesol (*p* < 0.01) and β‐eudesmol (*p* < 0.01) (Figure S7A). *Paenibacillus* (whose abundance was not displayed within the top 30−50 range) exhibited a negative correlation with atractylon (*p* < 0.001) and atractylodin (*p* = 0.11). Three strains bacteria of *Streptomyces* could all enhance the fresh weight of *A. lancea*, with *S. chromofuscus* and *S. mayteni* achieving statistical significance (*p* < 0.05) (Figure 4A,B). Both *S. cyaneochromogenes* and *S. mayteni* significantly decreased the content of hinesol (Figure 4C). Furthermore, these three strains notably reduced the content of β‐eudesmol (*p* < 0.05) but favored the accumulation of atractylon and atractylodin, with the latter achieving statistical significance (*p* < 0.05). These findings align with the results from the correlation heatmap between rhizosphere bacteria and volatile oils (Figure S7A). These aforementioned results indicate that *Streptomyces* facilitated the formation of MSA with a high ratio of (atractylon + atractylodin)/(hinesol + β‐eudesmol) (AA/HE) (Figure 4C). Conversely, although *P. alvei* within *Paenibacillus* inhibited the growth of *A. lancea* seedlings, it significantly boosted the content of β‐eudesmol (*p* < 0.001) and atractylodin (*p* < 0.001). And this elevated β‐eudesmol content was conducive to the formation of HBA with a low ratio of AA/HE (Figure 4C).

Within the endophytic core bacteria of the rhizome, *Rhodococcus* and *Ralstonia* exhibited significant negative correlations with hinesol (*p* < 0.01 and *p* < 0.001, respectively). *Sphingomonas* displayed significant negative correlations with both hinesol (*p* < 0.01) and β‐eudesmol (*p* < 0.01). Notably, although *Bradyrhizobium* was exclusive to HBA, it exhibited a positive correlation to some extent with all four volatile oils (Figure S7B). The verification results of microbial reconnection further showed that endophytic core bacteria positively influenced the fresh weight and total content of four volatile oils in *A. lancea*, particularly in boosting the content of atractylodin (Figure 4C). Three strains of *R. erythropolis* and two strains of *B. elkanii* significantly enhanced root fresh weight and volatile oil content. However, the same bacterial species exhibited strain‐specific effects on volatile oil composition. All *R. erythropolis* strains promoted atractylodin accumulation, but *R. erythropolis*1 and *R. erythropolis*2 inhibited hinesol content, while *R. erythropolis*3 did not affect hinesol, β‐eudesmol, or atractylon. Additionally, *B. elkanii*1 promoted hinesol, β‐eudesmol, and atractylodin, while *B. elkanii*2 only promoted atractylodin. Two *S. aquatilis* strains significantly enhanced atractylodin content, with *S. aquatilis*1 inhibiting hinesol. Two *R. solanacearum* strains significantly increased root fresh weight but had no significant impact on single plant fresh weight (Figure 4A,B). Both *R. solanacearum* strains promoted atractylodin, but *R. solanacearum*2 inhibited hinesol content. Among these endophytic core bacteria, seven strains increased the AA/HE ratio, favoring the formation of MSA, while the remaining six strains maintained or even decreased this ratio, conducive to the formation of HBA (Figure 4C). Ultimately, the distinct chemotype of *A. lancea* was shaped by the joint actions of rhizosphere and endophytic core bacteria and fungi (Figure S7C,D).

### Environmental factors driving chemotype formation in *A. lancea*

**Figure 5:**
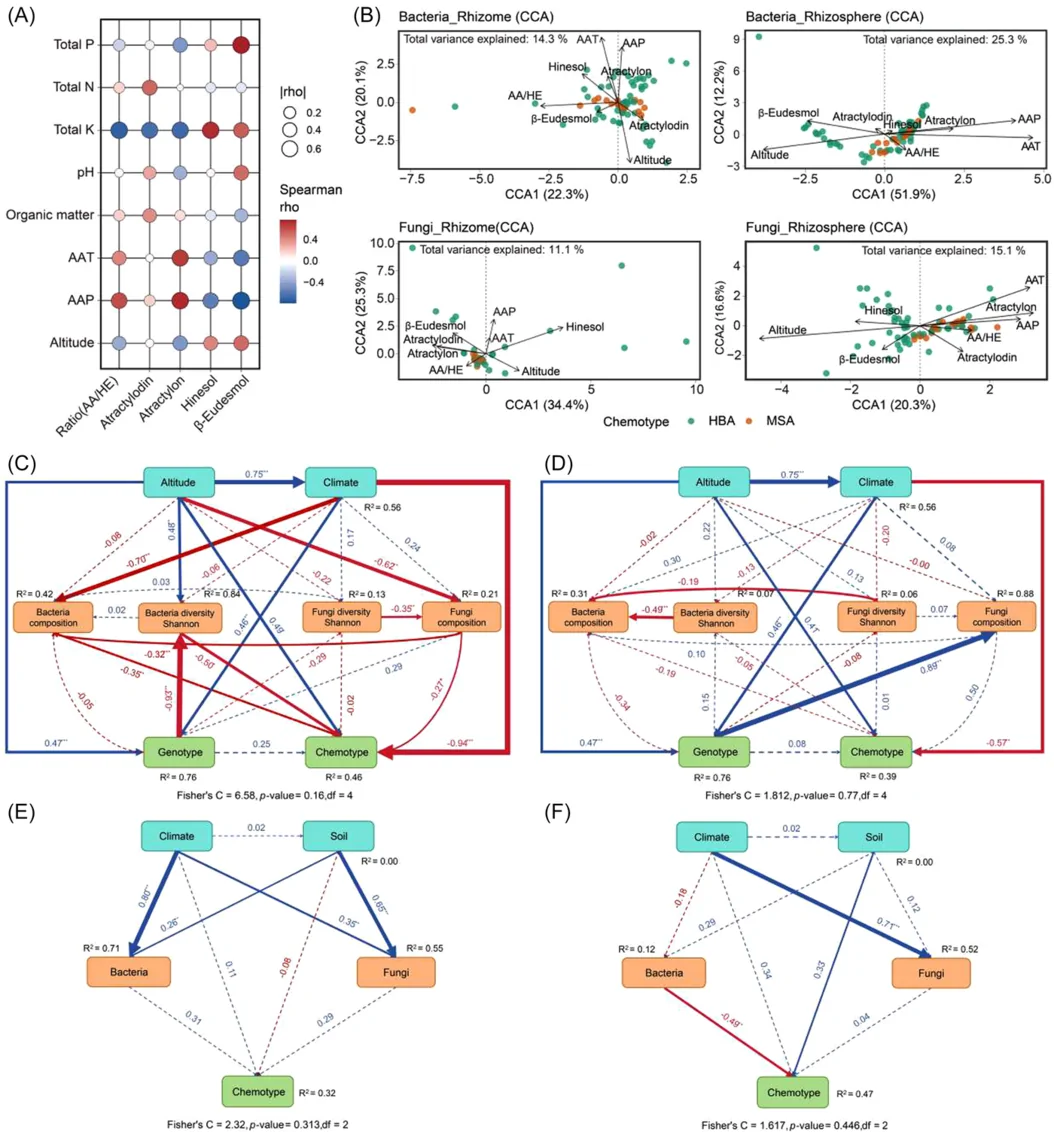
The relationships among soil factors, metabolites, and microbial communities, and the direct and indirect relationships between these factors and *A. lancea* genotypes and chemotypes. (A) Spearman correlation heatmap showing associations between environmental variables and metabolite ratios (e.g., AA/HE) or individual metabolites (e.g., atractylon). AAT: annual average temperature. AAP: annual average precipitation. (B) Canonical correspondence analysis (CCA) plots showing associations of rhizome/rhizosphere bacterial and fungal communities (points) with environmental variables (arrows: AAT, AAP), metabolites (e.g., hinesol, β‐eudesmol), and chemotype (HBA: green; MSA: orange). Total variance explained by CCA axes is indicated for each plot. Structural equation modeling analysis of the effect of altitude on chemotype formation through direct and indirect effects of climate, genotype, and microorganisms (C, D) or climate, soil, and microorganisms (E, F). The bacteria and fungi in C and E are rhizosphere microorganisms, while those in D and F are endophytic microorganisms. Blue and red solid arrows indicate statistically significant (*p* < 0.05) positive and negative correlations, respectively. The width of the arrows is proportional to the strength of path coefficients, as shown by the numbers adjacent to the arrows. *R* ^2^ indicates the proportion of variations in genotype and chemotype explained by the models. *p < 0.05, **p < 0.01, ***p < 0.001. The blue and red dotted arrows denote the absence of a statistical difference.

The soil microbial pattern is regulated by environmental conditions. Correlation analysis showed that high potassium (Total K) and phosphorus (Total P) significantly inhibited the accumulation of atractylodin and atractylon but promoted the synthesis of hinesol and β‐eudesmol (Figure 5A). In contrast, high temperature and precipitation exhibited the opposite trend: they suppressed hinesol and β‐eudesmol content but enhanced atractylon accumulation, with altitude exerting inverse effects to that of temperature and humidity (i.e., high altitude promoted hinesol/β‐eudesmol and inhibited atractylodin/atractylon). The canonical correspondence analysis (CCA) results indicated that environmental factors explained more variance in rhizosphere microbiota than that in endophytic microbiota (rhizosphere bacteria/fungi: 25.3%/15.1% vs. endophytic bacteria/fungi: 14.3%/11.1%), suggesting a stronger environmental regulation of rhizosphere communities (Figure 5B). Notably, altitude consistently counteracted temperature and precipitation: high temperature/precipitation primarily drove the associations between rhizosphere bacteria/fungi and atractylon, while high altitude strengthened the couplings between hinesol or β‐eudesmol and microbial communities. In summary, atractylodin and atractylon exhibited a “nutrient‐inhibited, climate‐promoted” pattern (suppressed by high K/P, promoted by high temperature/precipitation), whereas hinesol and β‐eudesmol showed a “nutrient‐promoted, climate‐inhibited” pattern (promoted by high K/P, suppressed by high temperature/precipitation, and enhanced by high altitude). Taken together, these results indicate that the rhizosphere environment demonstrates stronger regulatory roles in compound‐microbe interactions, as reflected by the higher explanatory power of environmental factors.

Following the variance inflation factor (VIF) analysis, out of the 27 environmental variables, five factors (VIF < 10) remained: altitude, average precipitation in April (prec4), May (prec5), and July (prec7); and average temperature in June (Tmean6). In the structural models depicting altitude and climate (Figure 5C,D), the chemotypes of *A. lancea* were influenced not only directly or indirectly by altitude but also exert more pronounced indirect effects by modifying the climate (A: 0.75, −0.94; B: 0.75, −0.57). The results from the Random Forest model revealed that the climate factor, prec7, played the most significant role in shaping the chemical types of *A. lancea*, with altitude being the second most important factor (Figure S8A,B). However, no direct correlation was observed between genotype and chemotype, suggesting that genotype does not entirely determine chemotype. The results from the Random Forest model further indicated that the importance of genotype was ranked after climate, altitude, and microbial factors, in terms of its influence on chemotype formation. Rather, altitude and genotype influenced chemotype formation by affecting rhizosphere bacterial diversity. Climate and altitude significantly affected the composition of bacteria and fungi in the rhizosphere, with a negative correlation between climate and bacteria and a negative correlation between altitude and fungi. While genotype exerted a stronger influence on the endophytic fungi, significantly affecting the fungal composition with a positive correlation.

In models of climate and soil, climate factors (prec5, prec7, and Tmean6) hold the utmost significance in determining chemotype formation, occupying the top spot (Figure S8C,D). Subsequently, rhizosphere bacteria composition, endophytic fungi composition, and soil properties (Total K) also contributed in this process. Climate exerted a significant influence on rhizosphere bacteria and fungi, and endophytic fungi, demonstrating a positive correlation (Figure 5E,F). Furthermore, soil chemical properties and endophytic bacteria directly affected the chemotype formation of *A. lancea* (Figure 5F), consistent with our experimental findings.

## 3 DISCUSSION

Genetic variation and environmental factors are key determinants in Dao‐di herbs' formation [26]. *A. lancea*, known for its significant polymorphism, exhibits considerable genetic variation both within species and across populations [27]. Over time, populations of *A. lancea* have undergone genetic differentiation due to long‐term environmental adaptation [7]. In the Maoshan area of Jiangsu, characterized by high temperatures, short dry seasons, and abundant precipitation, the unique ratio of volatile oils in *A. lancea* is likely a result of metabolic specialization to these specific environmental conditions. Temperature and precipitation, along with their interactions, are the main ecological factors influencing volatile oil content in *A. lancea*, with October having the greatest impact, followed by February and September [11]. High temperature, a limiting factor for *A. lancea*'s growth, reaches the peak value in the Maoshan area, significantly affecting Dao‐di herbs' formation.

The variation in volatile oils and genetic diversity in *A. lancea* follows distinct patterns. Many researchers have used chemical taxonomy to analyze the geographical variation of volatile oils in *A. lancea*, aiming to classify intraspecific variations, morphs, or chemotypes [1, 28, 29]. Takeda Osamu, a Japanese scholar, categorized *A. lancea* into three chemotypes, with the most influential classification into Dabie Mountain *A. lancea*, Hubei‐Anhui *A. lancea*, and Maoshan *A. lancea*, the latter including *A. japonica* [30, 31]. Our research identifies two primary chemotypes: MSA, which mainly comprises atractylon and atractylodin, and HBA, which mainly comprises hinesol and β‐eudesmol. MSA primarily includes MA, comprising wild and cultivated *A. lancea* from Jiangsu Province and cultivated *A. lancea* from Yuexi County, Anhui, while HBA includes both MA and SA. The interplay of genetic variation and environmental influences contributes to the phenotypic diversity of plants [32]. Our study shows significant differentiation in the volatile oil phenotype of HBA at the population level, despite minimal genetic differentiation. This suggests that environmental factors, rather than genotype alone, play a critical role in determining the chemotype of *A. lancea*. Japanese scholars [30, 33] have also confirmed this through transplant experiments, observing that non‐Dao‐di *A. lancea* transplanted to the Dao‐di area of MSA experienced a shift in volatile oil composition towards that of MSA after 2–3 years.

In recent years, the role of plant–microbe symbiotic systems in developing *A. lancea* resources has gained significant attention. Endophytic fungi in ALR enhance its environmental adaptability [34]. Yang et al. found that inoculating tissue‐cultured *A. lancea* seedlings with endophytic fungi promotes plant growth and medicinal active ingredient accumulation [35]. Zhou et al. studied cultivable endophytic bacteria in *A. lancea* leaves and identified their growth‐promoting potential for nitrogen fixation, phosphorus, and potassium solubilization [36]. Li et al. explored how *A. lancea* volatiles affect the growth of neighboring peanut plants in intercropping systems, showing the key role of microorganisms in plant interactions [37]. Chen et al. concluded that endophytic bacteria enhance the accumulation and the quality of volatile oil in ALR [38]. These findings highlight the significant impact of microorganisms on the chemical composition of *A. lancea*, particularly by influencing its volatile oil components. In this study, wild *A. lancea* tissue culture seedlings (MA and SA) were planted in native soil containing indigenous microorganisms (NMS and NHS) or sterilized soil (SMS and SHS). Our results revealed that native microorganisms or soil promote root growth and play a dominant role in chemotype formation, with minimal genotype influence. These results emphasize the crucial role of microorganisms in plant growth and chemical composition accumulation, providing a foundation for further studies on plant‐microbe interactions and optimization of cultivation and quality control.

Secondary metabolites are not only highly effective in treating human diseases but also crucial for plant growth, development, and defense. Furthermore, secondary metabolites function as vital communication signals, mediating complex interactions across different species. They facilitate exchanges between plants, between plants and microorganisms, and between plants and insects, thereby serving as key regulators and communicators in these intricate relationships [21, 39]. Research has revealed that microorganisms can significantly promote the accumulation of secondary metabolites in medicinal plants through various mechanisms. On one hand, certain microorganisms are capable of synthesizing plant hormones or modulating hormone levels within host plants, which in turn influences the synthesis and accumulation of secondary metabolites. On the other hand, these microorganisms can also activate the plant's immune system, trigger the expression of genes involved in metabolic pathways, and thereby significantly boost the content of secondary metabolites [40]. In this study, we discovered that the rhizosphere and endophytic core microorganisms associated with different chemotypes of *A. lancea* not only significantly modulated the levels of secondary metabolites but also demonstrated dual functionalities in promoting growth and providing biological control. This indicates that these microorganisms play an extremely important role in the metabolic regulation and the growth of *A. lancea*. *P. alvei* can induce the upregulation of amino acids, phytohormone, phenylpropanoids, flavonoids, and lipid compounds in sorghum, thereby endowing it with systemic resistance to *Fusarium pseudograminearum* [41]. Xie et al. found that *P. polymyxa* can effectively inhibit the growth of pathogenic fungi from *A. chinensis* root rot by producing volatile organic compounds, cellulases, and proteases [42]. One of the most notable characteristics of *Streptomyces* is its capacity to produce an extraordinarily diverse array of antibiotics [43]. Studies have also demonstrated that actinomycin D, produced by *S. cyaneochromogenes*, can inhibit the quorum‐sensing signals of pathogens, thereby preventing disease development [44]. Whole‐genome sequencing of *R. erythropolis* revealed that its genome harbors genes for the synthesis of antibiotics and iron carriers, endowing the bacterium with capabilities for promoting plant growth and exerting biocontrol functions. Additionally, the presence of quorum quenching signal genes in its genome plays a crucial role in inhibiting biofilm formation and virulence in pathogens [45]. Gao et al. found that *Rhodococcus sp*. AM201 can trigger the expression of genes related to reactive oxygen species (ROS), Ca²⁺ signal transduction, abscisic acid (ABA) signal inhibition, and plant root growth in *A. lancea* roots, and this bacterium is genetically similar to *R. erythropolis* [46]*. R. solanacearum* is a well‐known plant pathogen with a wide range of hosts, causing plant wilt disease that seriously affects the growth of crops such as tomatoes and potatoes [47]. These host plants can be divided into two categories: plants that can be caused diseases by *R. solanacearum*, and the other plants that cannot be caused diseases by *R. solanacearum* [48]. *A. lancea* obviously belongs to the latter, which may be related to the beneficial adaptive evolution of *R. solanacearum* to the host. Many *Sphingomonas* strains have been proven to promote plant growth and enhance plant stress resistance, mainly attributed to their abilities to fix nitrogen, dissolve phosphates, and produce plant growth hormones [49, 50]. For instance, disease‐resistant rice seeds with *S melonis* grown and passaged across several generations conferred resistance to disease‐susceptible phenotypes by producing anthranilic acid [51]. Dargiri et al. found that inoculating endophytic *S. aquatilis*, either alone or in combination with other microbes, can promote tomato growth by enhancing photosynthetic efficiency and increasing the content of phenolic compounds and proline [52]*. B. elkanii* is a symbiotic nitrogen‐fixing bacterium commonly known as rhizobia, capable of inducing root nodule formation in leguminous plants [53]. Research has found that *B. elkanii* can produce rhizotoxin, which regulates the interaction between plants and microorganisms by inhibiting *ACC* (1‐aminocyclopropane‐1‐carboxylate) synthase in ethylene (ET) biosynthesis in host roots [54]. In the rhizosphere and endophytic community of *A. lancea*, strains of *Burkholderia*, *Caballeronia*, and *Paraburkholderia* (collectively referred to as *B‐C‐P* due to classification confusion) are widely distributed [55, 56]. Previous studies have found that *Paraburkholderia* can regulate the content and composition of volatile oil in *A. lancea* [12]. *Burkholderia* is rich in genes encoding polyketide synthase and non‐ribosomal peptide synthase, which are involved in antibiotic production. *Paraburkholderia* not only directly inhibits pathogens or induces systemic resistance in plants to suppress diseases but also promotes plant growth and stimulates metabolic pathways. A prime example is the promoting effect of *P. xenovorans* on the volatile oil content of *A. lancea* [12].

Our study revealed that the functions of the core microbial community in *A. lancea* are closely associated with directly or indirectly stimulating plant systemic resistance and antagonizing pathogenic bacteria. This phenomenon may be linked to the presence of numerous potential pathogens (such as *Fusarium* and *Ralstonia*) in their rhizosphere and endophytic environments, despite these strains not causing disease under current conditions. Pathogens can trigger plant immune responses, including bursts of ROS, activation of mitogen‐activated protein kinases, induction of defense genes, and changes in hormone levels [47]. Research has shown that endophytic *Pseudomonas fluorescens* can trigger the ROS production in *A. lancea*, thereby converting anaerobic sesquiterpenes into oxygen‐containing sesquiterpenes and significantly increasing their content [36]. Additionally, endophytic *P. fluorescens* can activate gibberellin and ET signaling pathways by inducing hydrogen peroxide signaling, while ET further activates the ABA signaling pathway. Ultimately, gibberellin and ABA jointly promote the expression of key enzyme genes *HMGR* (3‐hydroxy‐3‐methylglutaryl‐CoA reductase) and *DXR* (1‐deoxy‐d‐xylulose 5‐phosphate reductoisomerase) in the sesquiterpene biosynthesis pathway, thereby enhancing the accumulation of volatile oils [57]. *Acinetobacter* [58] *sp*. promotes the accumulation of volatile oil in *A. lancea* through an endogenous ABA/salicylic acid‐dependent signaling pathway [57]. ET is an upstream signal of Jasmonic acid and salicylic acid, and a downstream signal of NO and H_2_O_2_ signaling pathways, and acts as an important signal mediating sesquiterpenoids biosynthesis of *A. lancea* induced by the endophytic fungus [59]. Jasmonic acid acts as a downstream signaling molecule in NO and H_2_O_2_‐mediated volatile oil accumulation induced by endophytic fungus and has a complementary interaction with the salicylic acid signaling pathway [60]. Ren et al. found that the endophytic fungus *Gilmaniella sp*. AL12 can induce Ca²⁺‐CAM (calmodulin) to regulate NO production and brassinosteroid concentration, thereby leading to the accumulation of volatile oil [61]. Phytohormones, such as salicylic acid (SA), jasmonates (JA), and ET, play crucial roles in the plant immune system, jointly regulating plant disease resistance and microbial balance [62]. This suggests that the plant response triggered by beneficial endophytic bacteria is similar to the immune response triggered by pathogens. Wang et al. revealed that co‐inoculation of endophytic fungi and bacteria can lead to reduced fungal colonization and inhibit plant defense responses [63]. Similarly, when two endophytic fungi are inoculated at different times, the first inoculated fungus affects the colonization of the later inoculated fungus [35]. These findings indicate complex interactions between rhizosphere and endophytic microbiota. In this study, we only investigated the effects of single strains on the plant growth and accumulation of volatile oils in *A. lancea* but did not explore the interactions between different strains. Additionally, although we predicted the functions of rhizosphere and endophytic core bacteria, we could not identify any metabolic pathways directly related to the promoting of plant systemic resistance. Therefore, further research is needed to elucidate the functions of these microorganisms and their interactions with each other.

The coevolution of plants and microorganisms offers extensive potential for microbial‐assisted plant breeding. Genetic mapping experiments have proven that in the model plant *Arabidopsis thaliana* [64], the staple crop maize (*Zea mays*) [65], and the cereal sorghum (*Sorghum bicolor*) [66], the heritable components of the microbiome composition on leaves and roots are governed by multiple loci. The structure of the microbiome differs across different plant species and among genotypes within the same species, and these variations are regulated by natural variation and artificial domestication [67, 68, 69]. Plant genotypes, particularly root architecture, exudate composition, and immune responses, significantly influence the composition and function of the rhizosphere microbiome. Key regulatory genes that affect the root's ability to recruit beneficial microbes have been identified and could serve as future breeding targets [70]. Genotypic differences in rice (*Oryza sativa*) have a highly significant effect on root‐associated microbial communities, as evidenced by the different root microbiomes associated with indica and japonica cultivars [71]. Plant domestication has altered the rhizosphere microbiome. During the domestication process from wild species to modern cultivars, crops have sought traits such as high yield and disease resistance, potentially losing some ability to interact with beneficial microbes [70]. Significant differences in the composition of the rhizosphere microbiome exist across different domestication stages of plants (wild species, local varieties, and modern varieties), providing a direction for reintroducing microbial interaction functions [72, 73]. Our results showed that the rhizosphere bacterial communities in wild and cultivated *A. lancea* were influenced by different genotypes (Figure S4D,H), while ALR exhibited the recruitment of the same microbiota (Figure 2C). Additionally, endophytic fungi exhibited genotype‐dependent distribution, with MA and SA being entirely distinct in the PCoA analysis (Figure S4G). Both the diversity and composition of rhizosphere and endophytic microbes exhibited genotype‐dependent regulation. Although the interaction mechanisms between genotypes and microbes need further investigation, this study provides guidance for the breeding of new *A. lancea* varieties through microbial assistance.

Phenotypic plasticity refers to the different phenotypic traits that the same genotype expresses in response to environmental factors. Both gene and environment contribute to phenotypic variation [74, 75]. *A. lancea*, a widely distributed species, exhibits strong environmental adaptability. Environmental factors, especially microorganisms, play a greater role than genotype in the formation of chemotype. Huang et al [76]. suggest that Dao‐di herbs' formation is influenced by geographical, historical, soil, climate, and human factors, with phenotype determined by genotype and habitat conditions. Biologically, Dao‐di herb formation is the result of genotype‐environment interactions, expressed as: phenotype = genotype + environmental modification [77]. Our findings indicate that abiotic factors, including altitude, climate, and soil, exert a direct influence on the chemotype of *A. lancea*, indirectly through its genotype and microbiome. However, genotype alone does not directly impact the chemotype; rather, it is the microorganisms that mediate its formation. The authenticity of traditional Chinese medicine stems from the unity of “genetics” and “environment.” With stable genetics, the ecological environment determines Dao‐di herbs' authenticity through complex plant–microbe interactions [25]. To understand the relationship between ecology and Chinese medicinal material authenticity, systematic research integrating plant growth, metabolites, and soil microecology is still required. This will support the creation of standardized, high‐quality Chinese medicine production methods.

## 4 CONCLUSION

This study examines the impact of biotic and abiotic factors, including altitude, climate, genotype, soil microorganisms, and soil chemical properties, on *A. lancea* chemotype formation. The results show that while altitude and climate drive genotype differentiation, genotypes alone do not determine chemotypes. Instead, soil microorganisms and chemical properties play a decisive role, with microorganisms exerting a stronger influence. Altitude, climate, and soil chemistry directly shape the genotype and indirectly affect chemotypes by influencing the microbiome. These findings support the hypothesis that phenotype results from genotype and environmental modification, offering insights for microbiological‐assisted breeding and targeted chemotype production of *A. lancea*.

## 5 METHODS

### Sample collection and processing

In August and September 2019, 78 rhizome samples and corresponding rhizosphere soil samples were collected from 16 wild and cultivated populations of *A. lancea* across five provinces in China: Jiangsu, Anhui, Hubei, Henan, and Shaanxi (Figure 1 and Table S1) using a shovel to carefully extract ALR, shaking off large soil pieces, and gently brushing off any remaining tightly adhered soil with a sterile brush. Each population had 4−6 replicates; 27 bulk soil samples were simultaneously collected by removing surface branches and fallen leaves from 4 to 5 locations (radius > 50 cm) where *A. lancea* did not grow and mixing soil from a depth of 5 cm. The bulk soil samples from each population were divided into three parts as replicates. All samples were transported to the laboratory on dry ice for further processing. For detailed processing methods, please refer to the supplementary method “Processing of ALR and soil samples, along with resequencing of wild samples.”

### Identification of the ITS sequence of *A. lancea* samples

The rhizome samples of *A. lancea* were sent to Beijing Ruibio Technology Co., Ltd. (Beijing) for DNA extraction and ITS sequence amplification. ITS primers [78]: ny47: 5'‐AACAAGGTTTCCGTAGGTGA‐3', ITS‐ASTI: 5'‐TGAGGACGCTTCTCCAGAC‐3'. PCR reaction system: 2.0 μL template DNA (100 ng/μL), 5.0 μL 10 × buffer (excluding Mg^2+^), 4.0 μL Mgcl_2_ (25 mM), 4.0 μL dNTPs (10 μM), 3.0 μL primer ny47 (10 μM), 3.0 μL primer ITS‐ASTI (10 μM), 5.0 μL DMSO, and 0.3 μL rTaq (5U/μL), to this ddH_2_O was added to prepare a 50 μL of the reaction system. The PCR reaction conditions were as follows: denaturation at 95°C for 5 min, denaturation at 95°C for 45 s, annealing at 56°C for 1 min, extension at 72°C for 1.5 min, for 38 cycles, followed by extension at 72°C for 10 min. The ITS sequence obtained from amplification has been uploaded to the NCBI with the sequence numbers OM622442–OM622519.

### Microbial DNA extraction, amplification, and sequencing

Microbial community genomic DNA was extracted from *A. lancea* rhizome or soil by using the PowerOil DNA Isolation Kit (Mo Bio Laboratories) according to the manufacturer's instructions. The quality of extracted DNA was checked on 1% agarose gel, and DNA concentration and purity were determined with NanoDrop2000 spectrophotometer (Thermo Scientific, United States). The primers 799F (5'‐ACMGGATTATACCKG‐3') [79]−1392R (5'‐ACGGCGGTGTRC‐3') [80] and 799F (5'‐ACMGGATTATACCKG‐3')−1193R (5'‐ACGCTCAT CCCCACCTTCC‐3') [68] were used to amplify the variable V5−V7 region of 16S rRNA in two steps. Primer ITS3F (5'‐GCATGAAGACGCAGC‐3')‐ITS4R (5'‐TCTCCCGCTTATTGATAGC‐3') [81] was used to amplify the ITS2 region. The reaction system was composed of 1 μL of the DNA template, 0.5 μL forward primers, 0.5 μL reverse primers, 0.25 μL bovine serum albumin, 12.5 μL 2× DreamTaq Green PCR Master Mix (Thermo Fisher Scientific), and ddH_2_O supplemented to 25 μL. Then, three technical replicates were set for each PCR reaction under the following reaction conditions: 95°C for 3 min; 95°C for 30 s, 27 cycles, 72°C for 45 s; 72°C for 10 min. The three technologies of a sample were repeatedly mixed and used as a PCR product, then separated by 2% agarose gel and purified by the Qiagen PCR purification kit (Kaijie Biotechnology Co., Ltd.). Purified amplicons were pooled in equimolar amounts and paired‐end sequenced on an Illumina Nextseq. 2000 platform (Illumina) according to the standard protocols by Majorbio Bio‐Pharm Technology Co. Ltd. The sequencing data have been uploaded to NCBI (project number: PRJNA806192).

### Amplicon sequence processing and analysis

After demultiplexing, the resulting sequences were quality filtered with fastp (0.19.6) [82] and merged with FLASH (v1.2.11) [83]. Then the high‐quality sequences were de‐noised using DADA2 [84] plugin in the Qiime2 (version 2020.2) [85] pipeline with recommended parameters, which obtains single‐nucleotide resolution based on error profiles within samples. DADA2 denoised sequences are usually called ASVs. Taxonomic assignment of ASVs was performed using the Naive Bayes consensus taxonomy classifier implemented in Qiime2 and the bacterial database Silva (Release 138, http://www.arb-silva.de) and the fungal database Unite (Release 8.0, http://unite.ut.ee/index.php). To assess the diversity and richness of bacterial and fungal communities, the Shannon and Chao indices were calculated using Mothur (v.1.30.1) [86]. PCoA was performed with QIIME (v.1.9.1) based on the Bray‐Curtis dissimilarity using Vegan package (v2.5‐3). Permutational multivariate analysis of variance (PERMANOVA) with 999 permutations was performed to test group differences in community composition. Differentially abundant taxa were identified using the lefser package [87], which implements the LEfSe algorithm (Wilcoxon rank test, FDR‐adjusted *p* < 0.05; LDA score > 2.0). Metagenomic functional profiles were predicted with PICRUSt2 [88] based on ASV tables, and functional annotations were obtained for KEGG orthologs (KOs), Enzyme Commission (EC) numbers, and MetaCyc pathways. Correlations between environmental variables and essential oils were assessed using Spearman's rank correlation. CCA was performed with vegan::cca(). Figures were generated with ggplot2 and pheatmap, and finalized in Adobe Illustrator.

### Evaluating the influence of native soil microorganisms and chemistry on chemotype formation in two genotypes of *A. lancea*

Wild *A. lancea* from Jiangsu and Henan belong to different genotypes and chemotypes. Tissue‐cultured seedlings from these regions were planted in peat soil mixed with either sterilized or nonsterilized native soil from their respective origins. This setup aimed to examine the influence of native soil microorganisms and chemistry on chemotype formation in the two genotypes. Detailed methods are provided in the supplementary material.

### Composition and functional verification of core microorganisms of two chemical types of *A. lancea*

The screening method for core microorganisms is based on the QIIME command “compute_core_microbiome.py” described by Pérez‐Jaramillo et al [89]. In this article, we define the rhizosphere and endophytic core bacteria refer to ASVs that are 100% present in all samples of MSA or HBA grouping. The rhizosphere and endophytic core fungi are defined as ASVs or species that are present in 80% of samples within MSA or HBA groupings. Notably, rhizosphere core fungi are identified at the ASV level, whereas endophytic core fungi are identified at the species level. The core rhizosphere and endophytic microorganisms of MSA and HBA were depicted using the pie function in R, displaying only the top 10 genera or species at the genus or species level, while the remaining genera or species were combined. Laboratory potting experiments verified the function of core microorganisms. Detailed methods are available in the supplementary material.

### Determination of four volatile oils in *A. lancea*

The freeze‐dried rhizome tissues of *A. lancea* were ground into powder using a ball mill (30 Hz, 2 min). Approximately 0.05 g of powder from each sample was weighed into a 2 mL Eppendorf tube, 1 mL of n‐hexane was added, and the mixture was sonicated at 60 Hz for 30 min. The supernatant was then centrifuged at 4°C and 5000×*g* for 5 min, filtered through a 0.22‐μm membrane (Sterivex, Millipore), and the volatile oil components in the filtrate were analyzed using gas chromatography‐mass spectrometry; detailed conditions are provided in the supplementary methods. The contents of hinesol, β‐eudesmol, atractylon, and atractyldin in each sample were quantitatively determined using standard curves (Table S3).

### Acquiring environmental factor data and piecewise structural equation modeling (pSEM) analysis

The altitude and latitude/longitude coordinates of the sampling points were recorded using a satellite positioning system (Table S1). Climate data for each sampling point were averaged from 3 to 4 nearby meteorological stations within <100 km. Data can be downloaded from the China Meteorological Administration (Tables S4 and S5). In addition, 27 bulk soil samples were sent to Beijing Shaoer Environmental Technology Co., Ltd. for pH, organic matter, total nitrogen, total phosphorus, and total potassium content analysis, following methods outlined in Table S6. pSEM was used to assess how altitude, climate, genotype, and soil chemical properties influence microbial community and chemotype in *A. lancea*. In the structural equation model, climate, soil, bacteria, and fungi all encompass multiple variables. Climate includes prec4, prec5, Tmean6, and prec7. Soil comprises organic matter, pH, total N, total P, and total K. Bacteria encompass bacterial diversity (Shannon index) and composition (PC1 within PCoA), while fungi encompass fungal diversity and composition. For each category with multiple variables, the first principal component (PC1) was used in the subsequent SEM analysis. Fisher's C test evaluated model fit, and revisions were made based on path significance (*p* < 0.05). Details of the pSEM fitting are described by Tian et al. [90]. The “piecewiseSEM,” “nlme,” and “lme4” R packages were used for pSEM.

### Data analysis

Data were processed in Microsoft Excel (Office 2019), and graphics were created using GraphPad Prism (v8.0.1). Sampling site distribution was mapped in ArcGIS (v10.2). The map of China (1:4 million) was obtained from the State Key Laboratory of Genuine Medicinal Materials, Chinese Medicine Resource Center, and the Chinese Academy of Traditional Chinese Medicine. PhyloSuite [91] was used to construct a phylogenetic tree for *A. lancea* samples and analyze relationships using maximum likelihood methods. Sankey diagram was performed using the ggsankey R package. The ranking of importance of factors, including altitude, climate, microorganisms, genotype, and soil chemical properties, in shaping the formation of chemotype was determined using the Random Forest package (v4.7‐1.1) in R (v4.4.3), with random seed fixed at 1234 for reproducibility [92].

## AUTHOR CONTRIBUTIONS

**Hongyang Wang**: Conceptualization; software; data curation; resources; writing—review and editing; writing—original draft; investigation; methodology; visualization; project administration. **Zheng Peng**: Validation; conceptualization; methodology. **Chengcai Zhang**: Writing—review and editing; visualization; software; supervision. **Chuanzhi Kang**: Supervision; conceptualization; methodology. **Yan Zhang**: Conceptualization; investigation; methodology. **Xiuzhi Guo**: Validation; software; data curation. **Yiheng Wang**: Visualization. **Guang Yang**: Conceptualization; investigation; visualization; data curation; formal analysis. **Zengxu Xiang**: Resources; investigation. **Li Zhou**: Project administration; supervision. **Zhixian Jing**: Visualization; software. **Dahui Liu**: Conceptualization; methodology; investigation. **Sheng Wang**: Conceptualization; methodology; investigation; resources. **Luqi Huang**: Conceptualization; project administration; supervision; funding acquisition. **Lanping Guo**: Funding acquisition; investigation; conceptualization; methodology; resources. All authors have read the final manuscript and approved it for publication.

## CONFLICT OF INTEREST STATEMENT

The authors declare no conflicts of interest.

## ETHICS STATEMENT

No animals or humans were involved in this study.

## Supporting information

The online version contains supplementary figures and tables available.

Figure S1 Phylogenetic tree based on the ITS fragment of the rhizome of *A. lancea* samples from different habitats.
Figure S2 Analysis of the relationship between climatic variations across different terrains and genotypic divergence.
Figure S3 Alpha diversity of bacterial and fungal communities across sample compartments.
Figure S4 PCoA of microbial communities by chemotype and genotype.
Figure S5 Pie charts of core fungal genera in rhizosphere and rhizome for MSA and HBA chemotypes.
Figure S6 Heatmap of rhizosphere core differential genera between chemotypes.
Figure S7 Spearman correlation heatmap between rhizosphere and endophytic microbiota of *A. lancea* and four major volatile oil components.
Figure S8 Random Forest was utilized to assess the ranking of importance of various factors in Piecewise SEM with respect to the formation of chemical types in *A. lancea*.

Table S1 Information of rhizome, rhizosphere soil, and control soil samples of *Atractylodes lance* from different habitats.
Table S2 The data displays the LEfSe analysis results of bulk soil, rhizosphere soil, and rhizomes between MA and SA (LDA score > 2, *p*adj < 0.05).
Table S3 Standard curves of hinesol, β‐eudesmol, atractylon, and atractylodin.
Table S4 Annual average temperature and monthly average temperature (unit: °C) from 2018 to 2019 of the sampling area.
Table S5 Annual average precipitation and monthly average precipitation (unit: mm) from 2018 to 2019 of the sampling area.
Table S6 Method for determination of soil pH, organic matter, total nitrogen, total phosphorus, and total potassium.

## Data Availability

All the data needed to evaluate the conclusions in the paper are presented in the paper and/or the supplementary materials. The 16S and ITS sequences were submitted to the SRA of the NCBI under accession numbers PRJNA806192 https://www.ncbi.nlm.nih.gov/bioproject/PRJNA806192/. The data and scripts used are saved in GitHub https://github.com/wanghybio/20250908iMetaOmics. Supplementary materials (methods, figures, tables, graphical abstract, slides, videos, Chinese translated version, and update materials) may be found in the online DOI or iMetaOmics http://www.imeta.science/imetaomics/.
