# Supplementary material for: The combined influence of microbiome and soil environment contributes to the chemotype differentiation in Atractylodes lancea
Source: IMetaOmics. 2025 Oct 9;2(4):e70058. doi: 10.1002/imo2.70058 (PMC12806005; doi:10.1002/imo2.70058)
Supplement: Supplementary file 1 — Figure S1 Phylogenetic tree based on the ITS fragment of the rhizome of A. lancea samples from different habitats. Figure S2 Analysis of the relationship between climatic variations across different terrains and genotypic divergence. Figure S3 Alpha diversity of bacterial and fungal communities across sample compartments. Figure S4 PCoA of microbial communities by chemotype and genotype. Figure S5 Pie charts of core fungal genera in rhizosphere and rhizome for MSA and HBA chemotypes. Figure S6 Heatmap of rhizosphere core differential genera between chemotypes. Figure S7 Spearman correlation heatmap between rhizosphere and endophytic microbiota of A. lancea and four major volatile oil components. Figure S8 Random Forest was utilized to assess the ranking of importance of various factors in Piecewise SEM with respect to the formation of chemical types in A. lancea. [file IMO2-2-e70058-s002.docx]

Supporting Information to

**The combined influence of microbiome and soil environment contributes to the chemotype differentiation in *Atractylodes lancea***

**Running title:** Microbiota Drives *A. lancea* Chemotype

Hongyang Wang^1,2,3#^, Zheng Peng^1,2,3#^, Chengcai Zhang^1,2,3#^, Chuanzhi Kang^1,2^, Yan Zhang^1,2,3^, Xiuzhi Guo^1,2^, Yiheng Wang^1,2^, Guang Yang^1,2^, Zengxu Xiang^4^, Li Zhou^1,2^, Zhixian Jing^1,2^, Dahui Liu^5^, Sheng Wang^1,2,3*^, Luqi Huang^1,2*^, Lanping Guo^1,2*^

^1^State Key Laboratory for Quality Ensurance and Sustainable Use of Dao-di Herbs, National Resource Center for Chinese Materia Medica, China Academy of Chinese Medical Sciences, Beijing 100700, China

^2^Key Laboratory of Biology and Cultivation of Herb Medicine, Ministry of Agriculture and Rural Affairs, Beijing 100700, China

^3^Dexing Research and Training Center of Chinese Medical Sciences, Dexing 334220, China

^4^College of Horticulture, Nanjing Agricultural University, Nanjing 210095, China

^5^Pharmacy Faculty, Hubei University of Chinese Medicine, Wuhan 430065, China

#These authors contributed equally: Hongyang Wang, Zheng Peng, Chengcai Zhang.

*Correspondence: [mmcniu@163.com](mailto:mmcniu@163.com) (Sheng Wang), [huangluqi01@126.com](mailto:huangluqi01@126.com) (Luqi Huang), [glp01@126.com](mailto:glp01@126.com) (Lanping Guo)

**METHODS**

**Processing of *A. lancea* rhizome and soil samples**

The ALRs were washed with tap water. Approximately 1 g of ALR was cut using a surgical blade for internal transcribed spacer (ITS) sequence amplification. Under sterile conditions, the second and third swollen rhizomes of wild *A. lancea*, near one end of the stem, were cut into 0.5 × 0.5 × 0.5 cm^3^ blocks and mixed in a 15 mL sterile centrifuge tube for later use. The morphological features of the rhizomes of both wild and cultivated ALR can be observed in the figure below. For cultivated ALR, 0.5 × 0.5 × 0.5 cm^3^ blocks were cut approximately 2 cm below the three buds in the middle of the rhizome and mixed in a 15 mL sterile centrifuge tube. Subsequently, the excised ALRs were rinsed three times with sterile distilled water. Next, they were treated with 70% ethanol for 10 min, followed by 10 min of ultrasonic treatment with 2.5% sodium hypochlorite. The tissues were then rinsed three more times with sterile distilled water and surface moisture was absorbed with sterile absorbent paper. The rhizomes and centrifuge tubes were frozen in liquid nitrogen and sent on dry ice to Shanghai Majorbio Bio-pharm Technology Co., Ltd. (Shanghai) for microbial total DNA extraction. The remaining rhizome samples were sealed in kraft paper envelopes and freeze-dried for volatile oil content determination.

**Resequencing of wild *A. lancea* samples**

The wild *A. lancea* samples utilized for resequencing were collected concurrently with the aforementioned wild samples, constituting the same batch. Each sample utilized exactly 1.5 μg of genomic DNA for the DNA sample preparation process. Following the guidelines provided by Illumina Inc., the Truseq Nano DNA HT sample preparation kit was used to create a sequencing library, with unique index codes assigned to each sample. Initially, the genomic DNA underwent ultrasonic shearing to attain a fragment size of ∼350 bp. Subsequent steps involved blunting the DNA fragment ends, A-tailing, and ligating them to adapters suitable for Illumina. This was followed by PCR amplification of the ligated fragments. Purification of the PCR products was carried out using AMPure XP from Beckman Coulter Inc., USA. The library size distribution was analyzed using an Agilent 2100 Bioanalyzer manufactured by Agilent Technologies Inc., USA. The libraries, once prepared, underwent sequencing on the Illumina HiSeq X Ten platform. Next, the fastq format raw reads were subjected to a comprehensive quality control regimen to ensure data integrity. This process involved the removal of low quality reads, identified as those containing ≥ 10% unidentified nucleotides, with > 10 adaptor-aligned nucleotides, allowing for a maximum of 10% mismatches, and with > 50% bases exhibiting a Phred quality score < 5. Additionally, putative PCR duplicates were eliminated.

After filtering for quality, the remaining paired-end reads underwent alignment using the BWA-MEM algorithm 0.7.8, employing the command mem -t 4 -k 32 –M. To mitigate potential mismatches, putative PCR duplicates were eliminated using SAM tools. Following alignment, SNPs were called using GATK. Only SNPs meeting stringent criteria with coverage depth > 8, minor allele frequency > 0.05, and missing data < 0.1 were kept for following analyses. To explore genomic perspectives of phylogenetic relationships, we created a neighbor-joining tree utilizing the raxmlHPC-PTHREADS command within the RAxML (v 8.0.19) software, with 100 bootstrap iterations for robustness assessment. ADMIXTURE v1.23 software was used to determine population genetic structure via the implemented expectation maximization algorithm. The phylogenetic tree was visualized using iTOL (Interactive Tree of Life; https://itol.embl.de), and the final layout and graphical elements were refined using Adobe Illustrator for clarity and visual consistency. The resequencing data employed in this research were sourced from a paper previously published by the authors in “Horticulture Research”, titled “Genome Resequencing Uncovers the Genetic Foundations of Population Evolution, Local Adaptation, and Metabolic Rewiring in the Rhizome of *Atractylodes lancea*” (doi: https://doi.org/10.1093/hr/uhae167).

**Cultivation of Seedlings of *A. lancea* with MA and SA genotypes**

The seeds of the wild *A. lancea* with the MA genotype were harvested from Jinniu Dong Mountain in Jintan City, Jiangsu Province. Meanwhile, those with the SA genotype were sourced from Nanshan in Checun Town, Song County, Luoyang City, Henan Province. Initially, the gathered seeds of both genotypes underwent surface sterilization using a specific concentration of mercuric chloride and sodium hypochlorite. Then the surface-sterilized seeds were placed on Murashige & Skoog (MS) medium to germinate surface-sterile plantlets. The aerial part of the approximately 2−3 cm tall plantlets were cut and cultured on solid MS medium (pH = 5.8) containing 30 g/L sucrose, 0.1 mg/L naphthalene acetic acid (NAA) and 1 mg/L 6-benzyladenine (6-BA) for vegetative propagation via tillering. Rooting was performed by culturing four-week-old vegetatively propagated *A. lancea* plantlets (approximately 4 cm tall) on the rooting medium, which was solid MS with 30 g/L sucrose and 0.5 mg/L NAA, for another four weeks. Once the seedlings of the two genotypes of *A. lancea* have successfully established their roots, they are individually transplanted into the soil needed for the experiment, which is contained in a small flowerpot (8 cm × 8 cm × 7.5 cm). After allowing the seedlings to grow in the soil for 9 to 10 days, white, slender roots can be observed emerging from the bottom of the flowerpot, signifying that the seedlings have successfully survived in the soil and are now ready for microbial reinoculation experiments.

**Evaluating the influence of native soil microorganisms and chemistry on the formation of chemotypes in two genotypes of *A. lancea***

To exclude abiotic factors like altitude and climate, we conducted a greenhouse experiment with indigenous microorganisms, exploring the interaction between genotype, microbes, and soil properties in chemotype formation. Soil samples from the native habitats of wild *A. lancea* were collected in Tangshan, Jiangsu (MS, E: 119.017283, N: 32.061882) and Song County, Henan (HS, E: 112.14538, N: 33.750052). MS serves as the soil conducive to the growth of MSA chemotype *A. lancea*, while HS is the soil that favors the cultivation of HBA chemotype *A. lancea*. After removing debris, the soil was sieved through a 2-mm mesh. A 350 g sample of MS or HS soil was mixed with 1 L of sterile water, shaken for 30 min, and combined with 2 kg of sterilized peat soil: vermiculite (3:1). This mixture was labeled as NMS and NHS. MA and SA were then cross-planted in NMS and NHS, resulting in four treatments: MANMS, MANHS, SANMS, and SANHS, each with 10 replicates. For sterilized soil, 350 g of MS or HS was treated with high-pressure steam (121°C for 30 min), then combined with 2 kg of sterilized peat soil: vermiculite (3:1), labelled as SMS and SHS. MA and SA were cross-planted in SMS and SHS, resulting in four treatments: MASMS, MASHS, SASMS, and SASHS, each with 10 replicates. The seedlings were cultivated in a plant culture room at 23 ± 2°C with 3000 lx light for 180 days, 12 h of light, and 12 h of darkness. After cultivation, 10 seedlings from each treatment were randomly selected, paired, and combined to form five replicates. The single plant fresh weight, rhizome weight, fibrous root weight, and root length were measured. Rhizomes were freeze-dried for volatile oil analysis.

**Functional validation of rhizosphere and endophytic core bacteria**

Since the formation of *A. lancea* chemotype by microorganisms does not depend on the genotype, we selected the MA seedling as the experimental material for the core bacteria function verification test here. *Streptomyces, Paenibacillus, Rhodococcus*, *Ralstonia*, *Sphingomonas*, and *Bradyrhizobium* are the rhizosphere and endophytic core microorganisms of *A. lancea*. *Streptomyces* is exclusively found in the rhizosphere core bacteria of MSA, whereas *Paenibacillus* and *Sphingomonas* are unique to the rhizosphere bacteria of HBA. *Rhodococcus*, *Ralstonia*, and *Sphingomonas* are present in the rhizomes of both chemical varieties of *A. lancea*, whereas *Bradyrhizobium* serves as both a rhizosphere core bacterium in MSA and HBA and an endophytic core bacterium in HBA. *S. chromofuscus*, *S. cyaneochromogenes*, *S. mayteni*, and *P. alvei* were isolated from the rhizosphere and rhizome of *A. lancea* and stored in the State Key Laboratory for Quality Ensurance and Sustainable Use of Dao-di Herbs. The 16S rRNA sequences corresponding to *Rhodococcus*, *Ralstonia*, *Sphingomonas*, and *Bradyrhizobium*, obtained through the use of the primer pair 799F_1193R, were extracted and compared within the NCBI database. The results indicated that they successful matches to *R. erythropolis*, *R. solanacearum*, *S. aquatilis*, and *B. elkanii*, respectively. Hence, the core rhizosphere bacteria, namely *S. chromofuscus*, *S. cyaneochromogenes*, *S. mayteni*, and *P. alvei*, along with the endophytic core bacteria *R. erythropolis*, *R. solanacearum*, *S. aquatilis*, and *B. elkanii*, were chosen for functional verification. *R. erythropolis* (Re1, Re2, Re3) were purchased from the Guangdong Provincial Microbial Culture Collection Center (preservation numbers: GDMCC 1.359), China Marine Microbial Culture Collection Management Center (preservation numbers: MCCC 1K00528), and China Agricultural Microbial Culture Collection Management Center (preservation numbers: ACCC41030), respectively. *R. solanacearum* (Rs1 and Rs2) and *B. elkanii* (Be2) were purchased from Zhili Zhongte (Wuhan) Biotechnology Co., Ltd., with storage numbers bio-23041, bio-107061, and bio-85261. *B. elkanii* (Be1) was acquired from the China Type Culture Collection Center (accession number CCTCC HB 20081039). *S. aquatilis* (Sa1 and Sa2) were purchased from Zhengzhou Jingsiwei Chemical Co., Ltd., with storage numbers bio-34564 and bio-096789. Microorganisms were cultured according to their specific needs: *S. chromofuscus*, *S. cyaneochromogenes*, *S. mayteni*, *R. erythropolis*, and *S. aquatilis* in trypticase soy broth (TSB), *R. solanacearum* in 2,3,5-triphenyl tetrazolium chloride (TTC), *B. elkanii* in yeast mannitol broth (YMB), and *P. alvei* in potato dextrose broth (PDB).

The aforementioned strains were individually cultivated in suitable liquid media at 37°C in a constant-temperature shaking incubator at a speed of 200 rpm for 48 h to yield microbial fermentation broth. Bacterial culture solutions were adjusted to a concentration of 10^−6^ CFU, and each *A. lancea* seedling was inoculated with 10 mL of bacterial suspension. The control groups were inoculated with 10 mL of sterile TSB, TTC, YMB, and PDB, respectively. Each treatment had 6 MA replicates and was cultured in an artificial climate chamber with a light intensity of 3000 lx, 12 h of light, and 12 h of darkness. After 40 days of microbial treatment, seedlings were carefully removed from the soil, washed with tap water, and the fresh weight and root length were measured. The six seedlings from each treatment were randomly divided into three groups (three replicates) and freeze-dried to constant weight. The dry weight was recorded, and the samples were used for volatile oil analysis.

**Determination of four main volatile oils of *A. lancea* seedlings**

Freeze-dried seedling roots were ground into a fine powder using a high-throughput tissue grinder (frequency: 60 Hz, time: 90 s). Approximately 0.1 g of powder was weighed and placed in a 2 mL centrifuge tube (Eppendorf). To this, 400 μL of n-hexane was added and thoroughly mixed. The mixture was then treated at 60 Hz for 30 min in an ultrasonic cleaner. Following this, the sample was centrifuged at 4°C and 5000 *× g* for 5 min using a low-temperature centrifuge. The supernatant was filtered through a 0.22 μm microporous membrane (Sterivex).

Gas chromatography-mass spectrometry was used to analyze the volatile oil components in the filtrate. The GC system was a Trace 1310 series, equipped with a TR-5 ms column (30 m^3^ × 0.25 mm i.d., DF = 0.25 mm) and a TSQ8000 mass spectrometer (Thermo Fisher Scientific). A 1 μL injection was made, with helium as the carrier gas at a flow rate of 1 mL/min and a split ratio of 20:1. The temperature program was as follows: initial temperature at 120°C for 2 min, then raised from 120°C to 240°C at 5°C/min, followed by a hold at 240°C for 5 min. The detector temperature was set to 350°C.

Mass spectrometry conditions included an electron ionization source temperature of 230°C, electron energy of 70 eV, and an interface temperature of 240°C. The mass scanning range was 40–500 m/z.


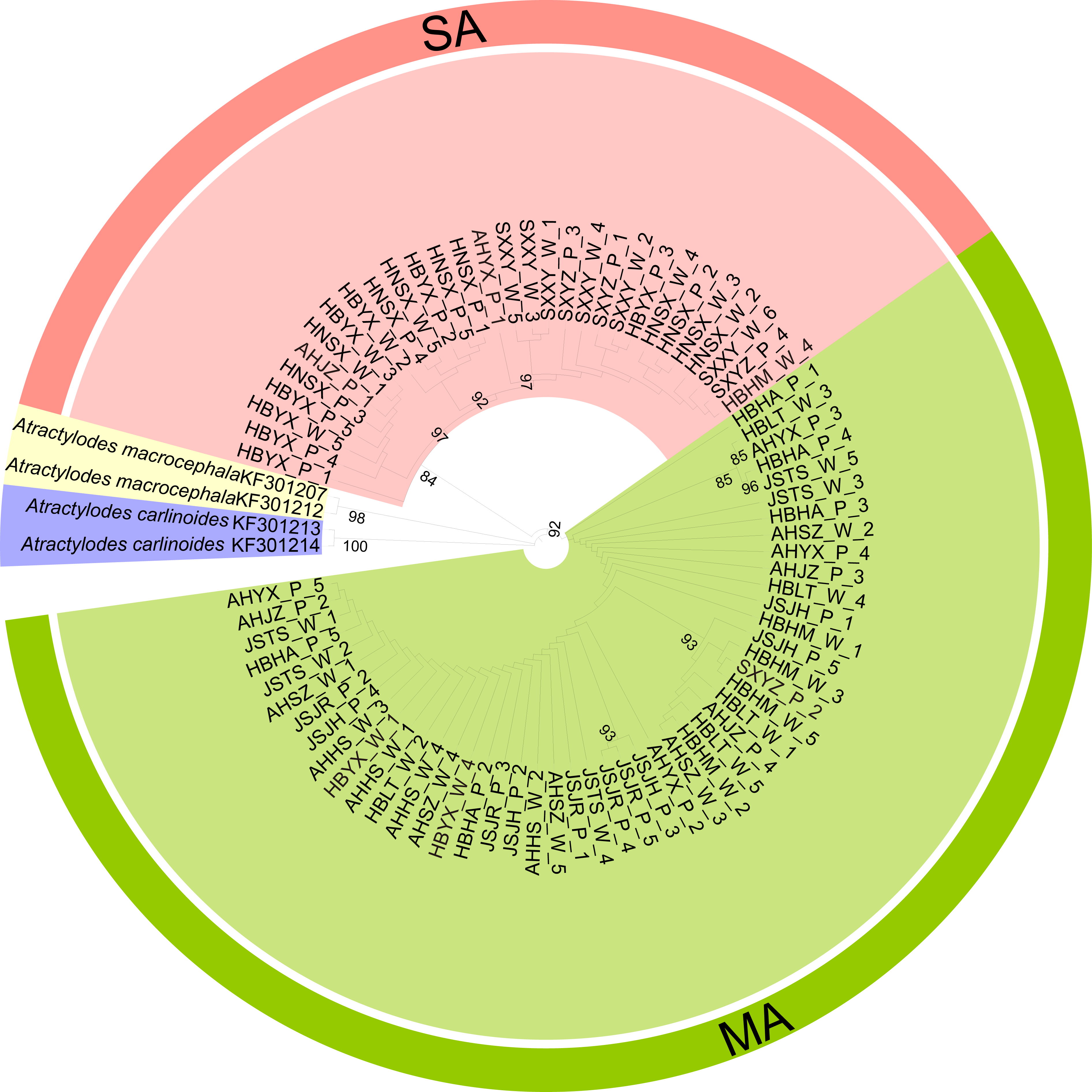


**Figure S1** **Phylogenetic tree based on the ITS fragment of the rhizome of** ***A. lancea* samples from different habitats.** On the branches of the evolutionary tree, the posterior probability values of maximum Likelihood autocorrelation inference are marked in the sequence. *A. macrocephala* and *A. carlinoides* are the outgroups of the phylogenetic tree. Samples featuring pink and green fonts were isolated from the local populations and excluded from subsequent analysis.


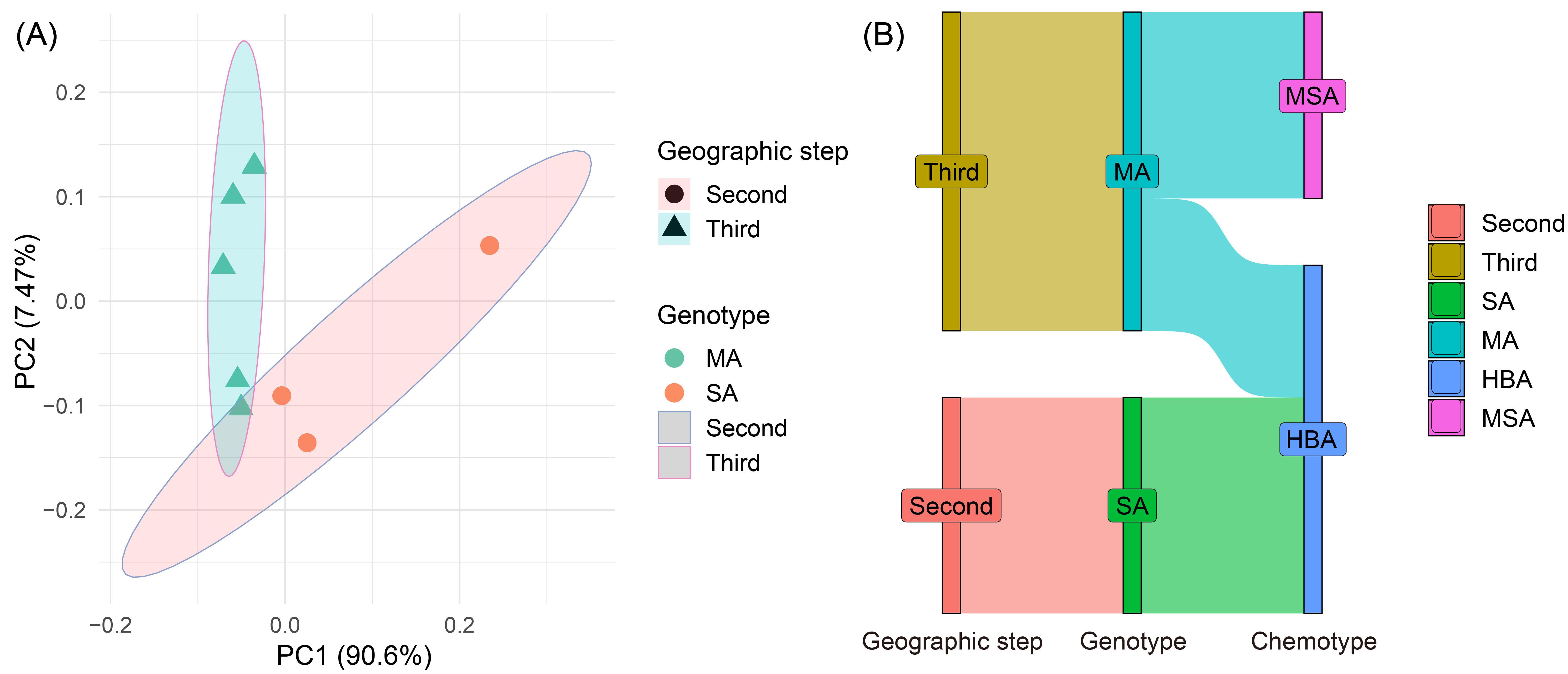


**Figure S2 Analysis of the relationship between climatic variations across different terrains and genotypic divergence.** (A) Principal component analysis (PCA) of climatic variables across the sampling sites. The proportion of variance explained by each principal component is indicated in parentheses on the axes. Permutational multivariate analysis of variance (PERMANOVA) with 999 permutations was performed to test group differences in terrain-induced climate (*p* < 0.001). (B) Sankey diagram of associations between China’s geographical gradients, genotypes, and chemotypes. Sankey diagram illustrating hierarchical relationships among China’s geographical gradients (*e.g.*, Third and Second regions), *A. lancea* genotypes MA and SA, and chemotypes HBA and MSA. Nodes are color-coded by factor (brown: the Third step, red: the Second step, teal: MA, green: SA, magenta: MSA, blue: HBA), with flow width proportional to sample size, representing sample distribution associations across levels.


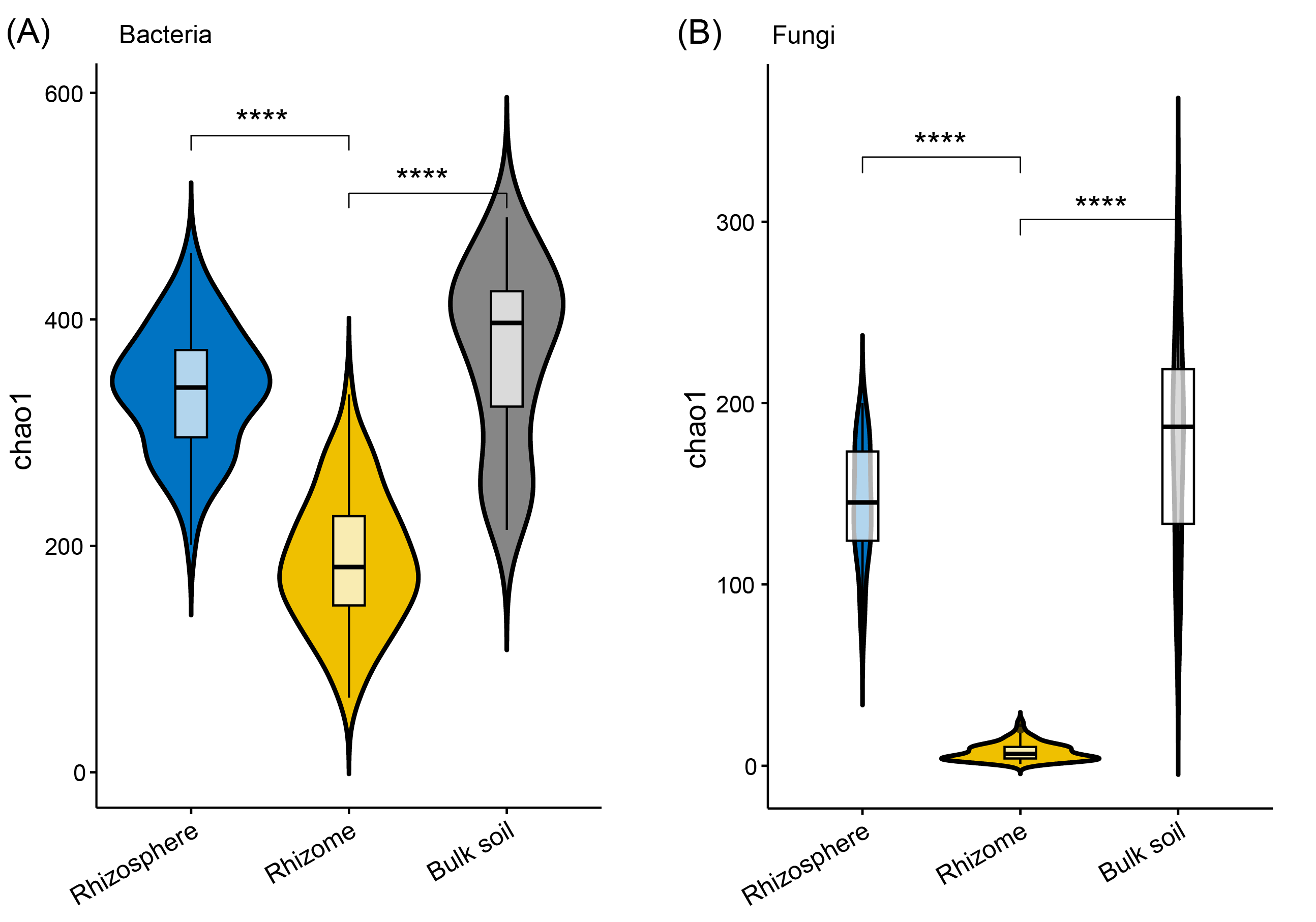


**Figure S3** **Alpha diversity of bacterial and fungal communities across sample compartments.** Boxplots showing Chao1 indices (indicating species richness) of bacterial (A) and fungal (B) communities in rhizosphere (blue), rhizome (yellow), and bulk soil (gray) samples. Significance was determined by Kruskal-Wallis test with Wilcoxon rank-sum post-hoc test (*****p* < 0.0001). Data are presented as boxplots with median (horizontal line), interquartile range (box).


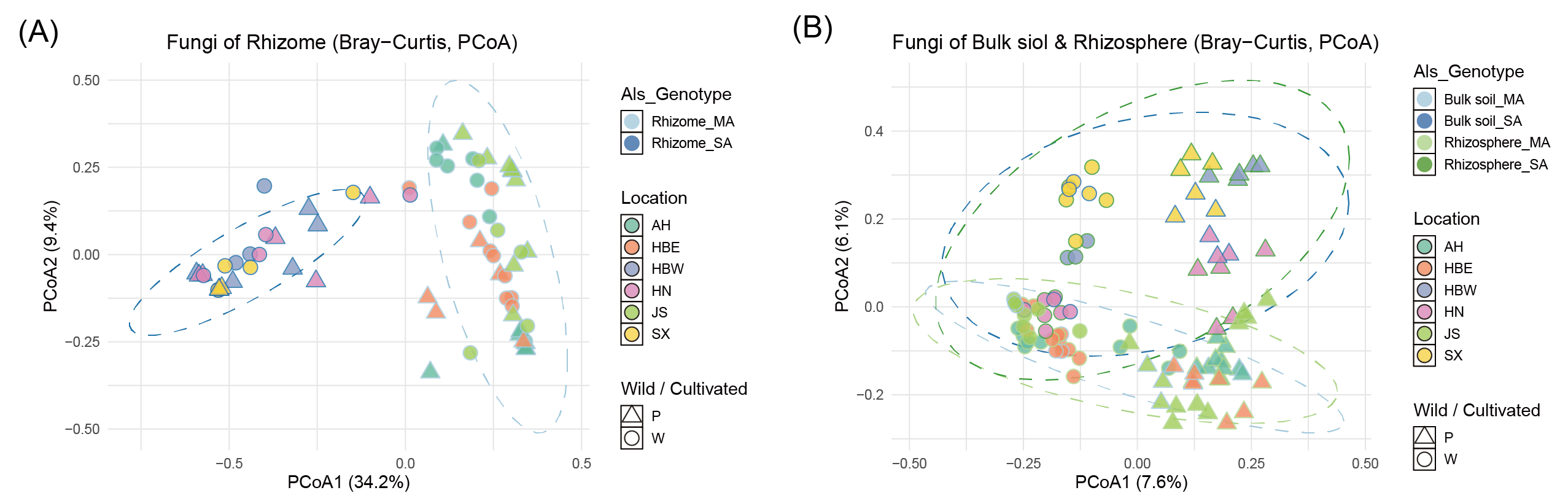


**Figure S4 PCoA of microbial communities by chemotype and genotype.** Bray-Curtis PCoA plots showing fungal (A and B) community structure. The fungal rhizome (A) and fungal bulk soil/rhizosphere (B) communities were separated by genotype (MA/SA). Ellipses represent 95% confidence intervals; axes show variance explained by PCoA1/2.


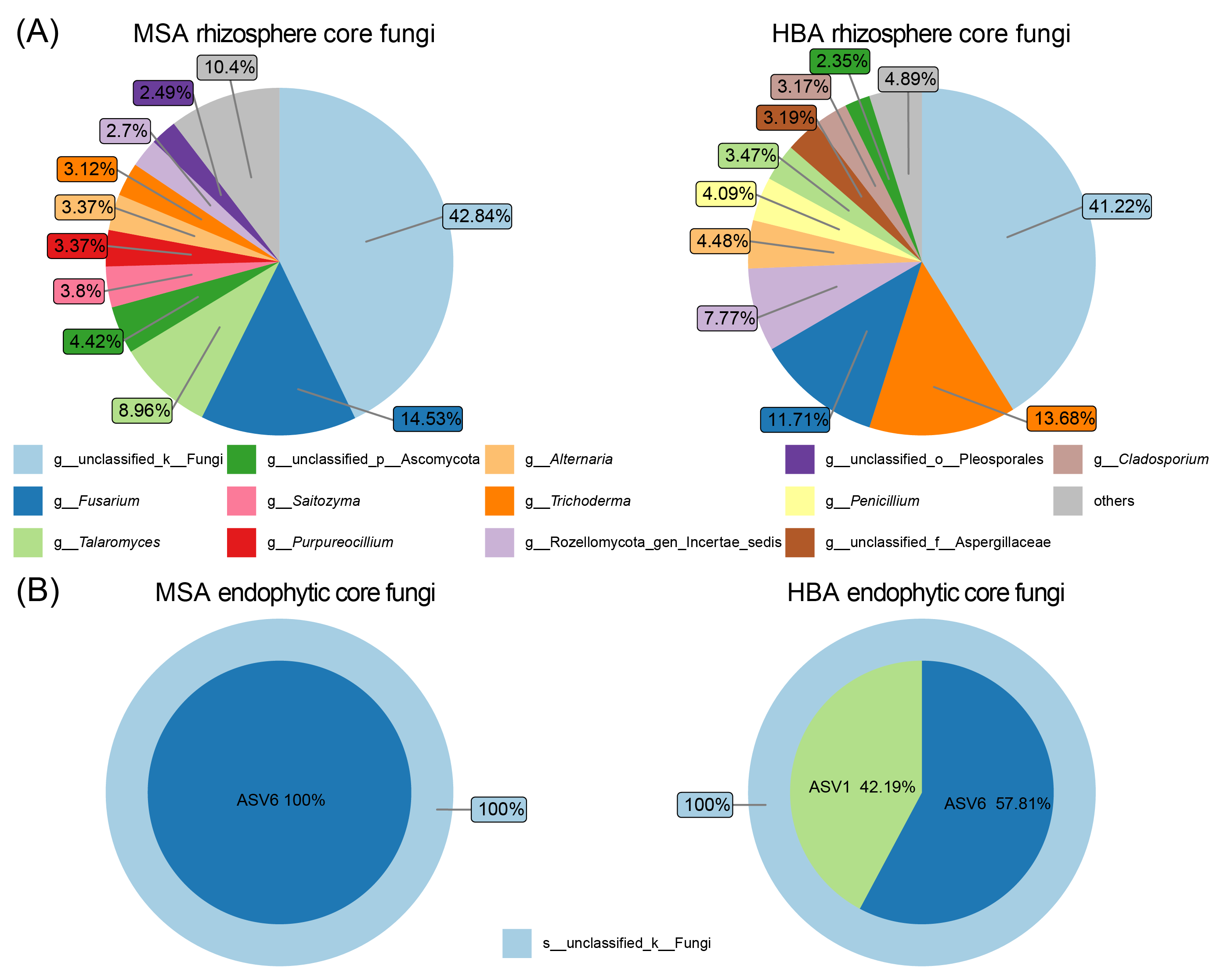


**Figure S5 Pie charts of core fungal genera in rhizosphere and rhizome for MSA and HBA chemotypes.** (A) Rhizosphere core fungi at the genus level. (B) Endophytic core fungi at the species level.


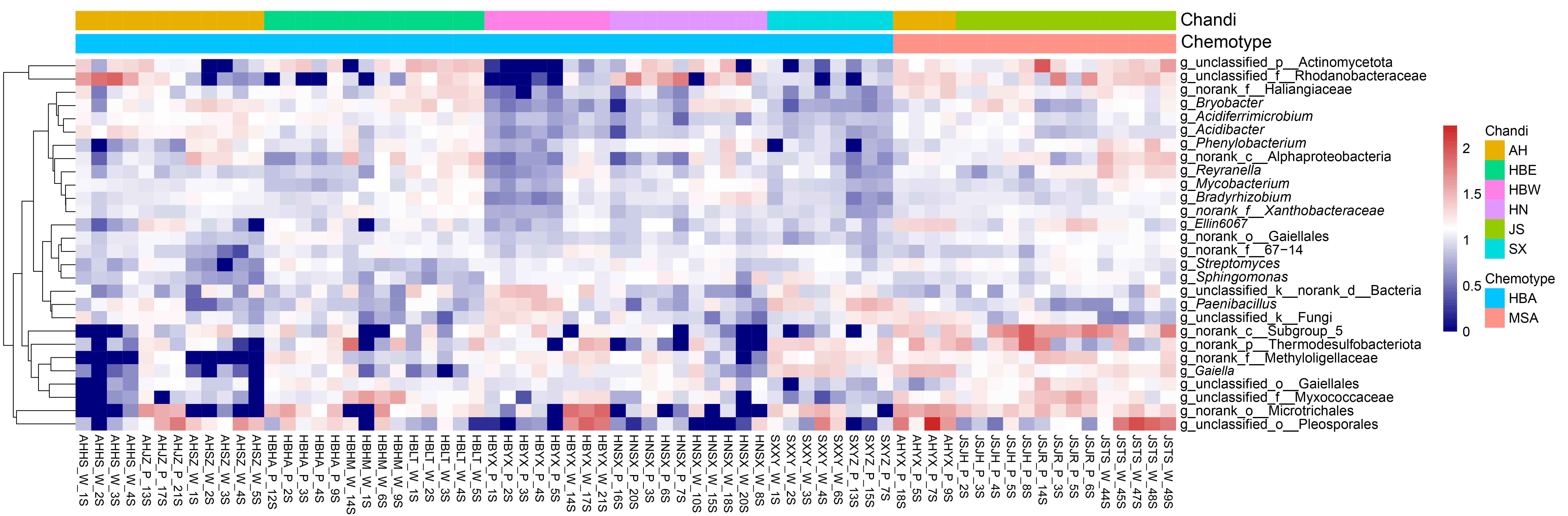


**Figure S6 Heatmap of rhizosphere core differential genera between chemotypes.** Heatmap showing row-normalized relative abundance of core differential bacterial and fungal genera between HBA and MSA chemotypes (rows: genera; columns: samples). Color intensity indicates normalized abundance (blue: low; red: high). Top annotations denote sample location (AH, HBE, HW, HN, JS, SX) and chemotype (HBA: light blue; MSA: red).


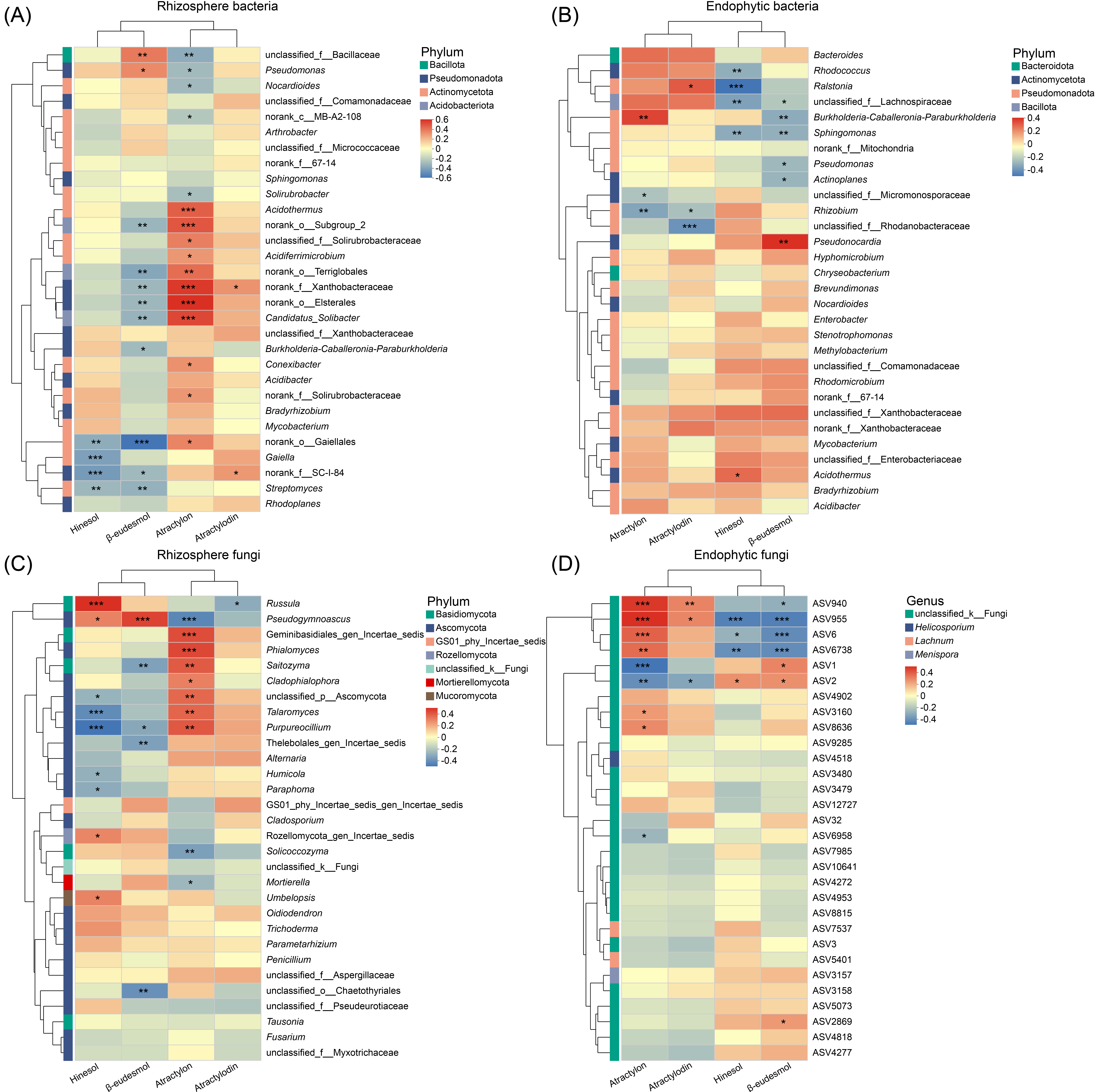


**Figure S7 Spearman** **correlation heatmap between rhizosphere and endophytic microorganisms of *A. lancea* and four major volatile oil components.** Heatmap of the correlation analysis between rhizosphere (A) and endophytic (B) bacteria and four types of volatile oils. Heatmap of the correlation analysis between rhizosphere (C) and endophytic (D) fungi and four types of volatile oils. * 0.01 < *p* ≤ 0.05, ** 0.001 < *p* ≤ 0.01, *** *p* ≤ 0.001.


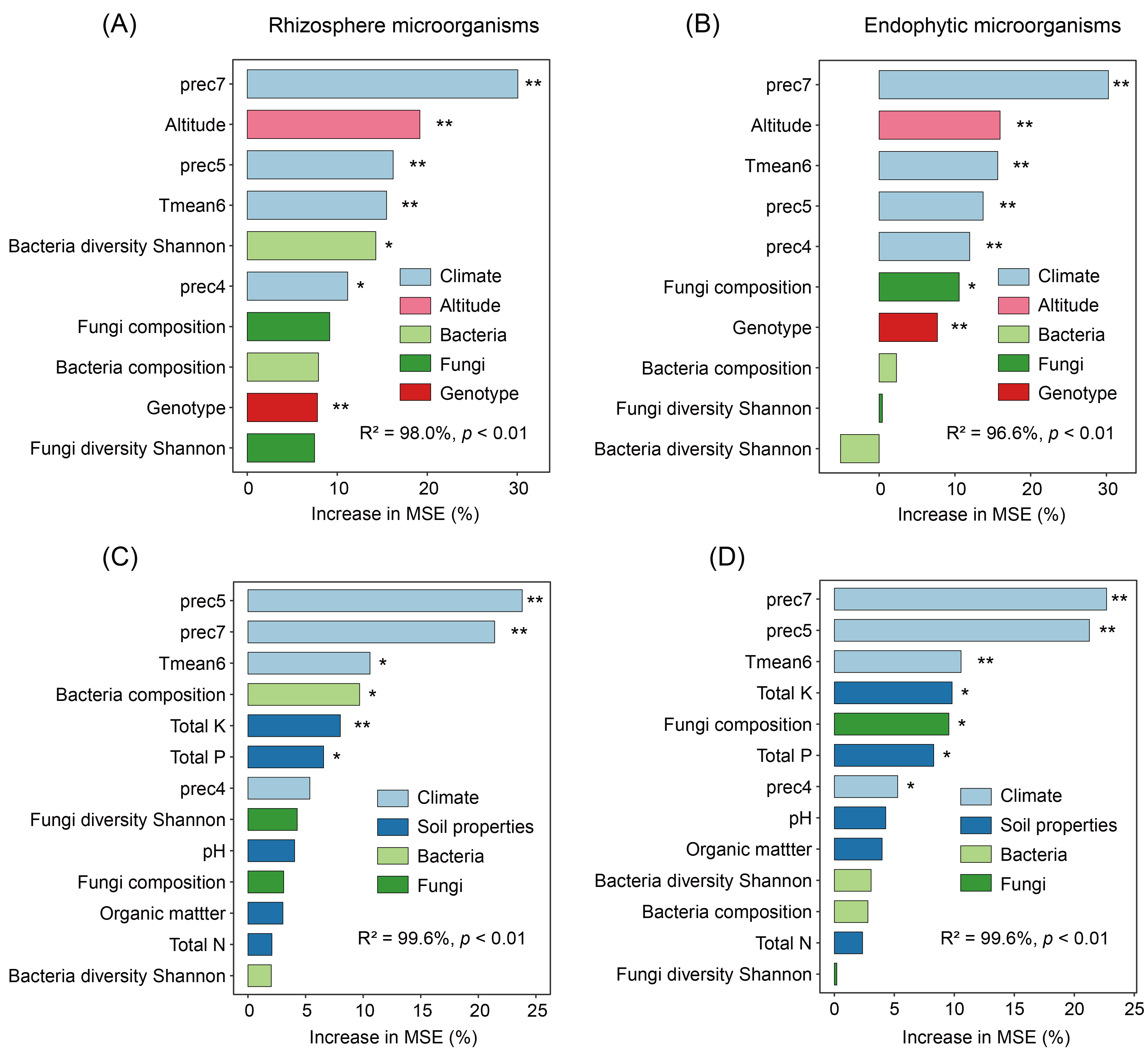


**Figure S8 Random Forest was utilized to assess the ranking of importance of various factors in Piecewise SEM with respect to the formation of chemical types in *A. lancea*.** The ranking of the importance of altitude, climate, genotype, and microbiota (A and B), or climate, soil, and microbiota (C and D), in the formation of chemotypes is presented in two models. * *p* < 0.05. ** *p* < 0.01.
